# A donor-acceptor integrated polymer for efficient organic solar cells

**DOI:** 10.1126/sciadv.aec1922

**Published:** 2026-04-08

**Authors:** Lunbi Wu, Xinkang Wang, Maggie Ng, Qingqi Song, Sha Liu, Ruijie Ma, Liwen Hu, Liangbin Xiong, Zhi Xing, Yao Li, Jiaying Wu, Ming Zhang, Shengjian Liu, Yue-Peng Cai, Man-Chung Tang, Feng Liu, Junwu Chen, Gang Li, Tao Jia, Fei Huang

**Affiliations:** ^1^ School of Optoelectronic Engineering, Guangdong Polytechnic Normal University, Guangzhou 510665, P. R. China.; ^2^ Dongguan Key Laboratory of Interdisciplinary Science for Advanced Materials and Large-Scale Scientific Facilities, School of Physical Sciences, Great Bay University, Dongguan, Guangdong 523000, P. R. China.; ^3^ Institute of Polymer Optoelectronic Materials and Devices, State Key Laboratory of Luminescent Materials and Devices, South China University of Technology, Guangzhou 510640, P. R. China.; ^4^ Institute of Materials Research, Tsinghua Shenzhen International Graduate School, Tsinghua University, Shenzhen 518055 P. R. China.; ^5^ Department of Electrical and Electronic Engineering, Research Institute for Smart Energy (RISE), Photonic Research Institute (PRI), The Hong Kong Polytechnic University, Hong Kong, China.; ^6^ Hangzhou International Innovation Institute, Beihang University, Hangzhou 311115‌, P. R. China.; ^7^ College of Chemistry and Materials Science, Gannan Normal University, Ganzhou 341000, P. R. China.; ^8^ Advanced Materials Thrust, Function Hub, The Hong Kong University of Science and Technology (Guangzhou), Nansha, Guangzhou 511400, P. R. China.; ^9^ School of Chemistry and Chemical Engineering, Frontiers Science Center for Transformative Molecules, In-situ Center for Physical Science, Center of Hydrogen Science, Shanghai Jiao Tong University, Shanghai 200240, P. R. China.; ^10^ School of Chemistry, Guangzhou Key Laboratory of Materials for Energy Conversion and Storage, Key Laboratory of Electronic Chemicals for Integrated Circuit Packaging, South China Normal University (SCNU), Guangzhou 510006, P. R. China.

## Abstract

Here, a donor-acceptor integrated polymer, PQIC, featuring a rigid π-conjugated framework, is reported, in which a Y-type small-molecule acceptor is covalently fused into a polymer donor backbone. PQIC exhibits balanced bipolar charge transport, reduced defect density, and high electroluminescence efficiency. When incorporated as a third component, it facilitates charge percolation, concurrently weakens electron-phonon coupling and lowers defect-state density, thereby alleviating recombination losses. As a result, PQIC-based ternary organic solar cells achieve a power conversion efficiency of 20.81% (third-party certified at 20.60%). In addition to high efficiency, the devices exhibit excellent stability, thick-film tolerance, and scalability, retaining ~85% of their initial efficiency after 2000 hours of maximum power point tracking, delivering 19.11% with a 300-nanometer-thick active layer, and reaching 19.78% for 1–square centimeter devices. These results highlight the potential of PQIC-based ternary systems for advancing organic solar cells.

## Teaser

A bipolar polymer enables efficient and stable organic solar cells with high thick-film tolerance.

## INTRODUCTION

**Fig. 1.**
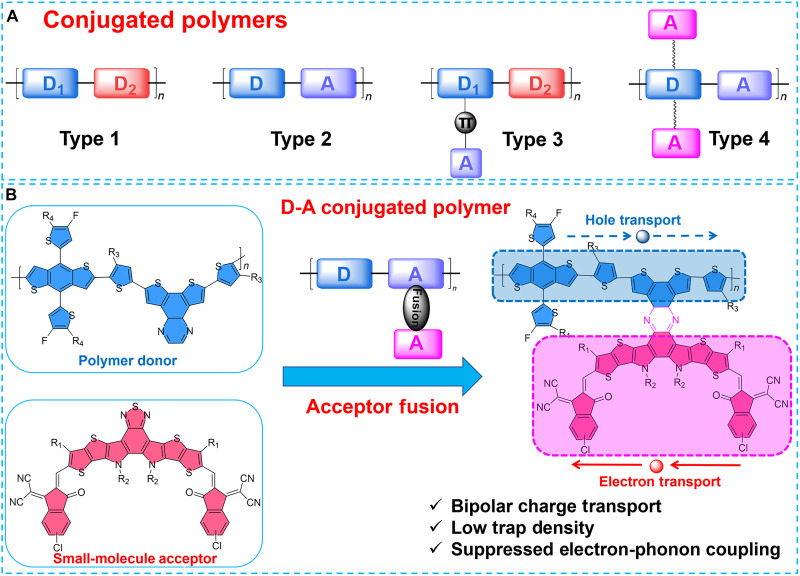
Molecule structure design. (**A**) Representative design directions of conjugated polymers. (**B**) Molecular design of fused-ring donor-acceptor integrated polymer and the corresponding characteristics.

Conjugated polymers, with their tunable energy levels, efficient charge transport, mechanical flexibility, and solution processability, have emerged as key semiconductors in optoelectronic applications such as organic solar cells (OSCs), organic light-emitting diodes, and organic field-effect transistors, with their pivotal role in OSCs driving rapid progress and enabling recent efficiency breakthroughs beyond 20% (*1*–*15*). To meet the performance requirements of OSCs, various conjugated polymers have been developed, and four representative design directions are briefly summarized (Fig. 1A): (i) donor-donor (D-D) polymers (*16*–*19*), composed exclusively of electron-rich donor units, which generally exhibit high hole mobility but offer limited tunability of energy levels and absorption spectra; (ii) donor-acceptor (D-A) polymers (*20*–*23*), incorporating alternating donor and acceptor moieties along the backbone, which allow precise bandgap engineering and frontier orbital alignment and therefore dominate current high-performance OSCs; (iii) acceptor-pended push-pull copolymers (*24*, *25*), in which pendant electron-accepting groups are introduced as side chains, enhancing intramolecular charge transfer, narrowing the bandgap, and improving molecular packing; and (iv) double-cable conjugated polymers (*26*–*28*), typically featuring a conjugated D-A backbone covalently tethered to electron-accepting side chains, ensuring efficient charge separation and transport. Despite these advances, the currently used conjugated polymers still suffer from intrinsic drawbacks—such as low molecular order, high defect density, and strong electron-phonon coupling—mainly arising from the inherent molecular vibrations of organic materials, which continue to limit device efficiency and stability of OSC compared with traditional photovoltaic (PV) technologies such as crystalline silicon and perovskite-based counterparts (*29*–*33*). Rigid fused-ring backbones offer an effective means to potentially suppress molecular vibrations, making the development of such conjugated polymers a promising strategy to overcome these intrinsic limitations (*34*–*37*).

In this work, we present a fused-ring donor-acceptor integrated polymer (Fig. 1B), PQIC, designed within a conventional donor-acceptor (D-A) framework. In this design, a high-performance Y-type small-molecule acceptor is conjugation locked with a quinoxaline-fused unit through ring fusion, forming an enlarged acceptor block that is longitudinally integrated into a polymer donor backbone, thereby yielding a rigid and continuous π-conjugated skeleton. Because of this structural strategy, PQIC exhibits intrinsic ambipolar charge transport, with hole and electron mobilities both higher than 10^−3^ cm^2^ V^−1^ s^−1^. The extended fused-ring architecture of the molecular backbone can effectively suppress molecular vibrations and enhance molecular ordering, thereby reducing the density of defect states. Moreover, this structure can also serve to effectively suppress electron-phonon coupling (*38*). As a result, when incorporated as a third component into the active layer, PQIC enables more efficient charge transport and substantially suppresses recombination losses, yielding a power conversion efficiency (PCE) of 20.81% with a third-party certified value of 20.60%, which ranks among the highest reported for OSCs. Benefiting from PQIC‘s intrinsic role in stabilizing the blend morphology, together with its high charge-carrier mobility and the additional mobility enhancement induced by active-layer crystallization, PQIC-based ternary devices exhibit substantially improved operational stability, thick-film tolerance, and large-area performances. This study highlights a versatile molecular design strategy that can inspire the development of conjugated polymers toward practically viable OSCs.

## RESULTS

### Polymer design, synthesis, and characterization

**Fig. 2.**
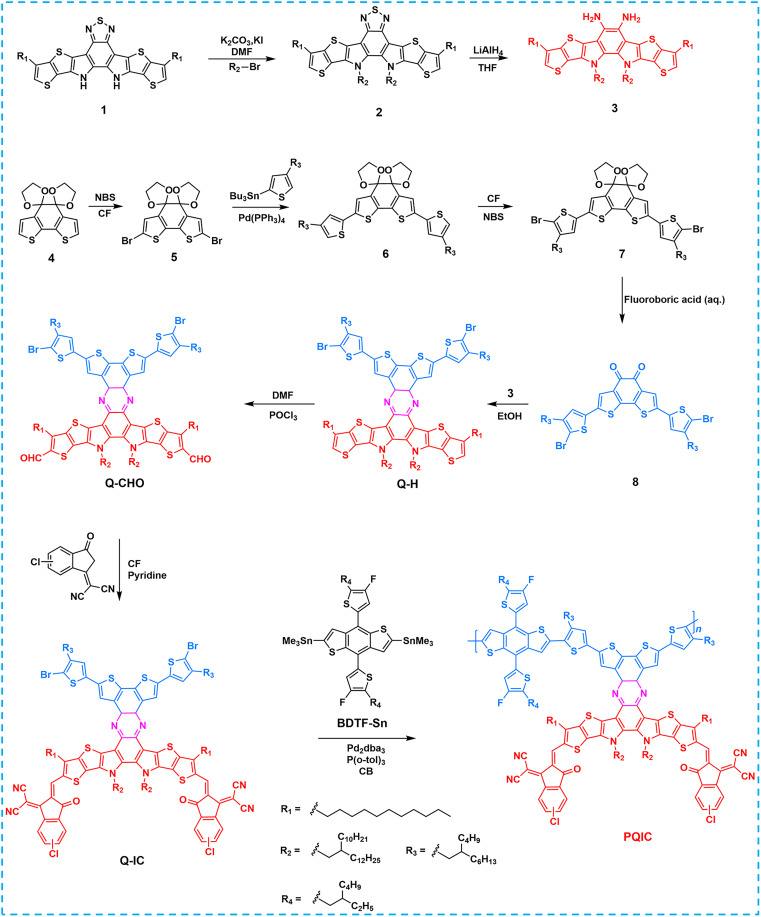
Synthetic routes. The synthetic routes of the intermediates and **PQIC**. DMF, *N*,*N*′-dimethylformamide; THF, tetrahydrofuran; CF, chloroform; aq., aqueous solution; CB, chlorobenzene; NBS, N-bromosuccinimide.

The chemical structures of PQIC are provided in Fig. 1B, respectively. This fused-ring D-A integrated conjugated polymer is constructed with a poly[(2,6-(4,8-bis(5-(2-ethylhexyl-3-fluoro)thiophen-2-yl)-benzo[1,2-b:4,5-b']dithiophene))-alt-5,5′-(6,9-bis(4-(2-butyloctyl)thiophen-2-yl)dithieno[3,2-f:2',3'-h]quinoxaline)] (PBQx-F)–based donor backbone (*39*), onto which 2,2′-((2Z,2′Z)-((13,14-bis(2-butyloctyl)-3,10-diundecyl-13,14-dihydrothieno[2′',3'':4',5']thieno[2',3':4,5]pyrrolo[3,2-f]thieno[2'',3'':4',5']thieno[2',3':4,5]pyrrolo[2,3-h]quinoxaline-2,11-diyl)bis(methaneylylidene))bis(5,6-difluoro-3-oxo-2,3-dihydro-1H-indene-2,1-diylidene))dimalononitrile (AQx-4F)–derived small-molecule acceptor units are linked via a rigid fusion (*40*, *41*). This design integrates extended fused-ring motifs across the molecular framework with the aim of suppressing molecular vibrations. Moreover, by structurally aligning its backbone with polymer donors and its side chains with Y-series small-molecule acceptors, this polymer may intrinsically function as a highly effective morphology compatibilizer. Figure 2 shows the synthetic routes of PQIC. Compounds **2**, **3**, and **4** were synthesized according to previously reported procedures (*27*, *42*). Compound **4** was subsequently subjected to a sequence of bromination, Stille coupling, a second bromination, and deprotection reactions, affording intermediate **8** (*42*, *43*). Coupling intermediate **8** with compound **3** through a quinoxaline ring–forming reaction afforded the key intermediate **Q-H**, which was transformed into the polymerizable monomer **Q-IC** by sequential Vilsmeier formylation and Knoevenagel condensation. Last, **Q-IC** was copolymerized with the distannylated comonomer (4,8-bis(4-(2-ethylhexyl)-5-fluorothiophen-2-yl)benzo[1,2-b:4,5-b’]dithiophene-2,6-diyl)bis(trimethylstannane) (**BDTF-Sn**) via Stille coupling to yield the target polymer **PQIC**. The chemical structures of all intermediates and the final polymer were unambiguously confirmed by ^1^H nuclear magnetic resonance (NMR), ^13^C NMR, mass spectrometry (MS), and high-temperature gel permeation chromatography (figs. S1 to S14). PQIC has a high molecular weight (*M*_w_ = 57.5 kDa) and exhibits excellent solubility in chloroform, chlorobenzene, and *o*-xylene, which is advantageous for its solution processability and compatibility with diverse solvent systems.

### Optoelectronic characteristics and molecular simulations

**Fig. 3.**
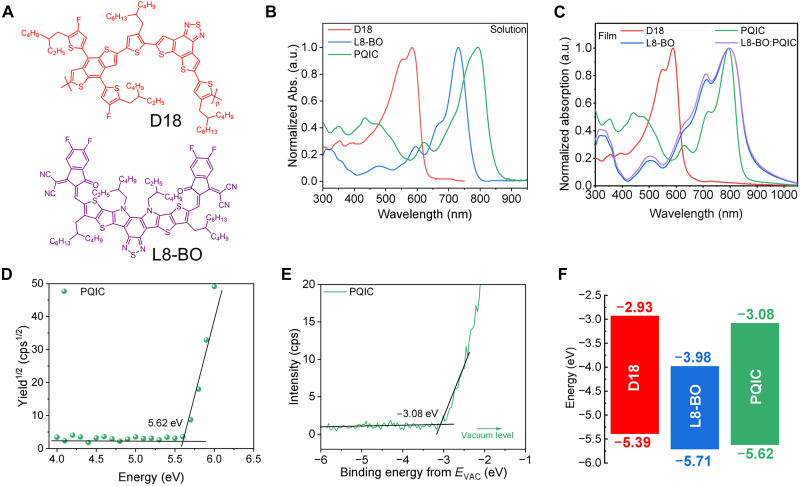
Chemical structures of photoactive materials and the related physical properties. (**A**) Chemical structures of D18 and L8-BO. The normalized absorption profiles in diluted solutions (**B**) and film states (**C**). Abs., absorption; a.u., arbitrary units. The PESA (**D**) and IPES (**E**) results of PQIC. (**F**) Energy level alignments of D18, L8-BO, and PQIC. cps, counts per second.

The chemical structures of D18 and L8-BO are illustrated in Fig. 3A. The ultraviolet-visible (UV-Vis) absorption spectra of D18, L8-BO, and PQIC in solutions and films are shown in Fig. 3 (B and C), respectively. From solution to film, D18 exhibits almost no spectral shift, whereas L8-BO shows a pronounced red shift, indicating improved molecular packing in the solid state. The nearly unchanged maximum absorption peak of PQIC from solution to film, together with the sharpening of the 716-nm peak, indicates that PQIC is already highly aggregated in solution and undergoes further ordering during film formation. In addition, the PQIC film shows a relatively narrow full width at half maximum (FWHM) without any broadening of the absorption peak, indicating suppressed molecular vibrations and implying a low defect density inherent to its rigid fused-ring structure (*44*, *45*). Besides, blending a small amount of PQIC with L8-BO not only sharpens the absorption peak at 716 nm but also induces a red shift of the absorption edge, indicating that PQIC may potentially promote the crystalline ordering of L8-BO. Furthermore, the energy level alignments were analyzed by combining photoelectron emission spectroscopy in air (PESA) and inverse photoelectron spectroscopy (IPES) measurements. As shown in Fig. 3 (D to F) and fig. S15, the derived highest occupied molecular orbital (HOMO) and lowest unoccupied molecular orbital (LUMO) energy levels of PQIC lie between those of D18 and L8-BO, which is expected to form a gradient energy-level alignment when PQIC is used in the active layer (Table 1).

**Table 1.** The optical properties and energy level properties.

Materials	λ_max_^sol.^ [nm]	λ_max_^film^ [nm]	λ_onset_^film^ [nm]	*E*_g_^opt *^ [eV]	*E*_HOMO_ ^†^ [eV]	*E*_LUMO_ ^‡^ [eV]
D18	550,580	551,588	633	1.96	−5.39	−2.93
L8-BO	731	796	878	1.41	−5.71	−3.98
PQIC	794	794	848	1.46	−5.62	−3.08

* $E_{g}^{\text{opt}}=1240/{\lambda_{\text{onset}}}^{\text{film}}$.

† Estimated from PESA measurements.

‡ Estimated from IPES measurement.

**Fig. 4.**
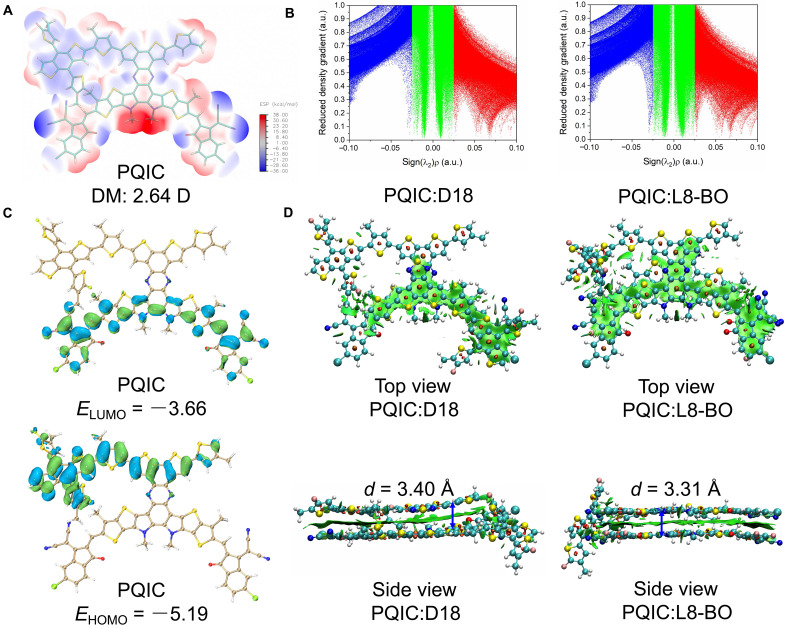
DFT calculation and molecular simulation results. (**A**) ESP distributions of PQIC. (**B**) Calculated HOMO and LUMO distributions. (**C**) The functions between IRI and Sign(λ_2_)ρ. (**D**) Weak interaction regions depicted by the independent gradient model based on Hirshfeld partition. DM, dipole moment; ESP, electrostatic potential.

To understand the electronic characteristics and intramolecular packing of PQIC, density functional theory (DFT) calculations were performed. As shown in Fig. 4A, the positive electrostatic potential is located around the thiophene and nitrogen atoms, while the negative potential is found near the cyano and carbonyl groups. The polymer donor backbone shows a neutral to slightly positive potential, whereas the acceptor segment features a neutral or positive core and negatively charged terminal groups. A relatively large dipole moment of 2.64 D was observed. The calculated HOMO and LUMO energy levels of the PQIC model are −5.19 and −3.66 eV, respectively, with the HOMO predominantly localized on the polymer backbone and the LUMO mainly distributed over the fused-ring small-molecule acceptor (Fig. 4B). Accordingly, charge transfer is likely to occur from the polymer backbone to the side-chain small-molecule acceptor. The interaction region indicator (IRI) versus Sign(λ_2_)ρ scatter plots reveal that both D18:PQIC and L8-BO:PQIC exhibit strong intermolecular interactions (Fig. 4C). The respective packing distances of D18:PQIC and L8-BO:PQIC pairs induced by the intermolecular interactions are calculated. As illustrated in Fig. 4D, the packing distances were determined to be 3.40 Å for D18:PQIC and 3.31 Å for L8-BO:PQIC. This suggests that PQIC is capable of establishing relatively compact intermolecular packing with both D18 and L8-BO (*46*).

### PV performance, charge generation, and recombination

**Fig. 5.**
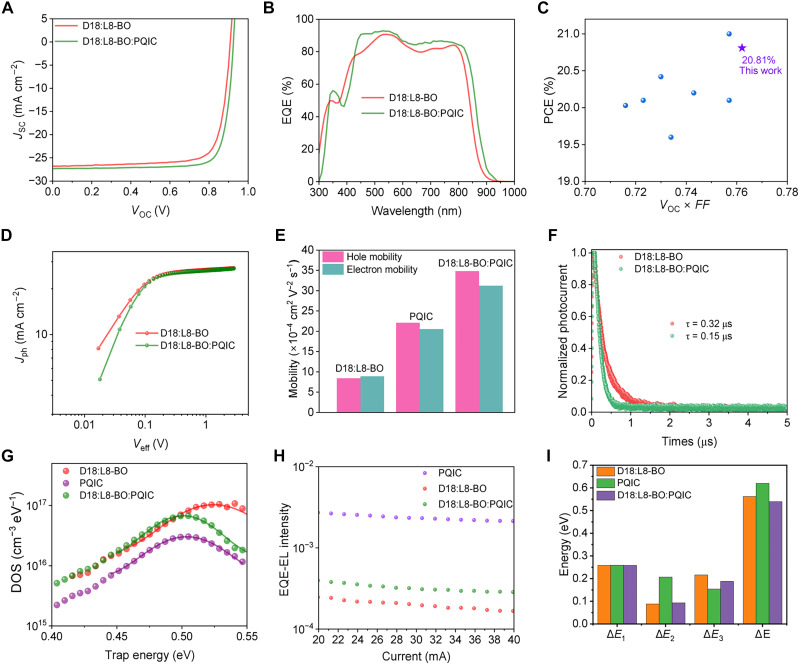
PV performance, charge generation, transport, and combination loss analyses. (**A**) *J-V* characteristics of binary and ternary OSCs. (**B**) EQE spectra. (**C**) The performance summary and comparison. (**D**) Photocurrent versus effective voltage (*J*_ph_-*V*_eff_) relationships. (**E**) Calculated hole and electron mobilities. (**F**) Transient photocurrent (TPC) curves. (**G**) Total density of states (t-DOS) curves. (**H**) EQE–electroluminescence (EL) results. (**I**) Summarized energy loss parameters.

The PV performance was assessed by fabricating OSC devices, with the current density-voltage (*J-V*) characteristics and external quantum efficiency (EQE) spectra presented in Fig. 5 (A and B) and fig. S16, respectively. The PQIC-only device exhibits a markedly low PCE, primarily due to its absolutely low short-circuit current density (*J*_SC_) and fill factor (*FF*), indicating its limited photoelectric conversion when used alone. A possible reason is that this molecular design strategy hinders the timely separation of charges due to the integration of donor and acceptor motifs into single fused-ring architecture. However, insights gained from the molecular property analysis prompted us to attempt ternary devices within high-efficiency system. The D18:L8-BO binary devices show an efficiency of 19.14%, with a *J*_SC_ of 26.8 mA cm^−2^, an open-circuit voltage (*V*_OC_) of 0.908 V, and an *FF* of 78.5% (Table 2). The ternary devices were optimized under identical processing conditions, with the PQIC content (relative to the donor mass) and film thickness systematically tuned. The introduction of PQIC into D18:L8-BO led to simultaneous improvement of all three PV parameters. The device achieves an optimal PCE of 20.81%, with a *J*_SC_ of 27.3 mA cm^−2^, a *V*_OC_ of 0.926 V, and an *FF* of 82.3%. This efficiency and fill factor rank among the highest reported values. Notably, a third-party certification verified an efficiency of 20.60% (fig. S17). The EQE spectra reveal that PQIC alone exhibits no photoresponse (fig. S16B); however, it substantially enhances both the intensity and spectral range of the photoresponse in ternary devices (Fig. 5B). In addition, the integrated current density values derived from the EQE spectra validate the accuracy of the *J*-*V* measurement, with the *J*_SC_ mismatch remaining below 5% for the optimized device. To highlight the effectiveness of our results, the correlation between *V*_OC_ × *FF* and efficiency was plotted, as presented in Fig. 5C and table S1. The purple star symbol, located in the upper-right corner of the plot, illustrates the superior performance achieved through our strategy, underscoring the great potential of fused-ring donor-acceptor integrated polymers in boosting OSC performance. We also explored the generality of PQIC in ternary systems. As shown in figs. S18 and S19 and tables S2 to S5, incorporating an appropriate amount of PQIC leads to clear efficiency enhancements in the PM8:L8-BO and PTQ10:Y6 systems, as well as in the D18:L8-BO system processed with *o*-xylene. These results collectively demonstrate the broad applicability of PQIC across different active-layer systems and processing solvents. To evaluate device scalability, large-area devices (1 cm^2^) were further examined. As shown in fig. S20, the ternary device achieves a higher PCE of 19.78% than the binary device (17.31%), with superior efficiency retention of 95.1% from small-area to large-area devices, compared to 90.4% for the binary system. This result may highlight the beneficial role of PQIC in improving the aggregation state of the blend solution and facilitating large-area processing (*47*).

**Table 2.** Device performances.

Active layer	*V*_OC_ [V]	*J*_SC_ ^*^ [mA cm^−2^]	*J*_SC_ *^†^* [mA cm^−2^]	*FF* [%]	PCE ^‡^ [%]
D18:L8-BO	0.908 [0.906 ± 0.004]^‡^	26.8 [26.9 ± 0.3]^‡^	24.9	78.5 [78.0 ± 0.5]^‡^	19.14 [18.85 ± 0.29]^‡^
D18:L8-BO:PQIC	0.926 [0.925 ± 0.003]^‡^	27.3 [27.4 ± 0.2]^‡^	26.4	82.3 [82.0 ± 0.4]^‡^	20.81 [20.61 ± 0.20]^‡^ 20.60*^§^*

* Obtained from *J-V* curves.

† Integrated from EQE spectra.

‡ Averaged PCE values are based on eight independent devices.

§ Efficiency certified by a third party.

To further elucidate why PQIC, despite exhibiting no intrinsic photoresponse, can markedly enhance the efficiency of ternary devices, we carried out additional device physics investigations. As shown by Fig. 5D, the photocurrent versus effective voltage (*J*_ph_-*V*_eff_) relationships are plotted to investigate electron generation process. The single-component device based on PQIC exhibits negligible charge generation capability (fig. S21). This result reveals that PQIC itself remains intrinsically inefficient, primarily due to perturbations in the charge-generation process. Moreover, the D18:L8-BO binary and D18:L8-BO:PQIC ternary devices could reach the saturated photocurrent density (*J*_sat_) at a low effective voltage region (*V*_eff_ ≈ 0.2 V). The ratio of *J*_sc_ to *J*_sat_ can be used to evaluate the exciton dissociation efficiency (*P*_diss_), and the ratio of the current density at the maximum power output point to *J*_sat_ can be used to assess the charge collection efficiency (*P*_coll_). The *P*_diss_/*P*_coll_ values for D18:L8-BO and D18:L8-BO:PQIC devices are 0.963/0.905 and 0.985/0.923, respectively. The improvements in both *P*_diss_ and *P*_coll_ for D18:L8-BO:PQIC devices indicate optimized exciton dissociation and charge collection processes.

The space-charge–limited current method was used to investigate the charge transport characteristic. As shown in Fig. 5E and fig. S22, neat PQIC exhibits efficient and nearly balanced ambipolar transport, with electron and hole mobilities of 2.05 × 10^−3^ and 2.11 × 10^−3^ cm^2^ V^−1^ s^−1^. These results confirm the predicted bipolar character and the high carrier mobility imparted by the integrated fused-ring structure, which may facilitate both hole and electron transport pathways. Meanwhile, the binary devices exhibit hole and electron mobilities on the order of 10^−4^ cm^2^ V^−1^ s^−1^. Upon incorporating PQIC into the D18:L8-BO, PQIC substantially enhances the overall carrier mobilities. Specifically, the hole and electron mobilities are enhanced by more than threefold relative to the binary counterpart, mainly driven by both intrinsic charge-transport contributions and morphology optimization (table S6). The charge extraction and recombination are evaluated by transient photocurrent (TPC) and photovoltage measurements, as well. According to the plots and derived carrier lifetimes in Fig. 5F and fig. S23, more accelerated carrier extraction and suppressed recombination are obtained. The density of state (DOS) parameter are detected, as shown in Fig. 5G and table S7. PQIC exhibits a low trap density, primarily ascribed to its extended fused-ring architecture that effectively suppresses molecular vibrations. Therefore, when adding PQIC in D18:L8-BO host system, it can effectively reduce the overall trap density from 5.93 × 10^15^ to 2.91 × 10^15^ cm^−3^. By minimizing deep trap states within the matrix, the charge mobility is naturally increased.

Furthermore, the detailed energy losses of PQIC single-component, D18:L8-BO binary, and D18:L8-BO:PQIC ternary devices were evaluated using sensitive-EQE (s-EQE), electroluminescence (EL), and EQE-EL experiments (*48*, *49*). The s-EQE and EL spectra are shown in fig. S24, where the bandgaps were determined from the differentiated EQE spectra (fig. S25). The corresponding energy loss parameters are illustrated in Fig. 5I and summarized in table S8. As shown in Fig. 5H, PQIC demonstrates a notably high EL efficiency at the 2 × 10^−3^ level, which is 10 times higher than that of D18:L8-BO. This can be attributed to the low defect-state density and reduced disorder induced by PQIC, which effectively suppress nonradiative decay and promote radiative recombination (*50*). As a result, PQIC-based devices exhibit an absolutely low nonradiative recombination losses (Δ*E*_3_) of 0.154 eV (*51*). Upon incorporation into the active layer, PQIC effectively enhances the luminescence of the D18:L8-BO:PQIC system, thereby reducing Δ*E*_3_ from 0.216 eV to as low as 0.188 eV (Fig. 5I). To the best of our knowledge, this represents one of the lowest values reported at such an efficiency level, highlighting the effectiveness of our polymer design strategy.

## DISCUSSION

### Crystallization and molecular packing behavior

**Fig. 6.**
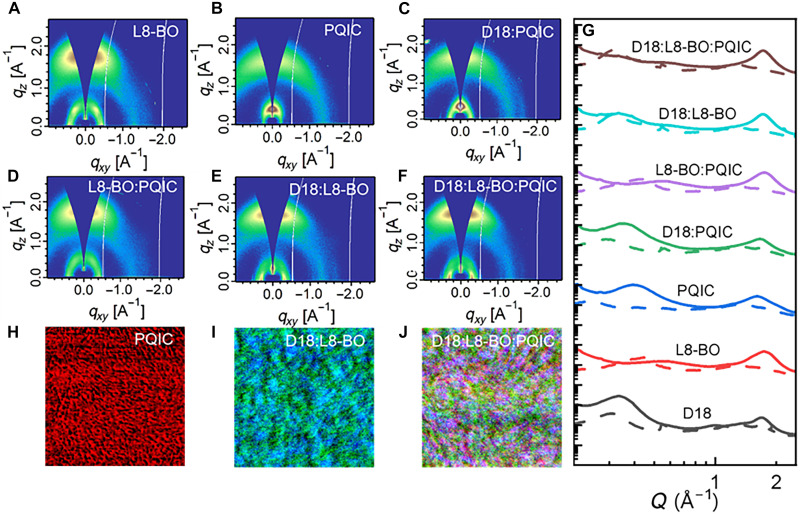
Morphology characterizations. (**A** to **F**) 2D GIWAXS patterns of binary and ternary blend films. (**G**) Related line-cuts. (**H** to **J**) Photoinduced force microscopy (PiFM) images for PQIC neat film, and two blend films.

The molecular packing behavior was systematically analyzed using the grazing incidence wide-angle x-ray scattering (GIWAXS) measurement, with the resulting two-dimensional (2D) scattering patterns presented in Fig. 6 (A to F) and fig. S26 and the corresponding in-plane (IP) and out-of-plane (OOP) line-cuts are shown in Fig. 6G. By comparing the data of pristine D18, L8-BO, and PQIC with their blend films (D18: PQIC and L8-BO: PQIC), it can be concluded that PQIC generally reduces the crystallinity of D18 while enhancing the face-on orientation of L8-BO. Moreover, when incorporating PQIC into D18:L8-BO, a notable enhancement in the overall crystallinity of the active layer was observed, as evidenced by the increased diffraction intensities in both lamellar and π-π stacking directions. In particular, the lamellar stacking *d*-spacing stays nearly unchanged at ~20 Å from the D18:L8-BO to the D18:L8-BO:PQIC, suggesting preservation of the fundamental polymer backbone packing distance. However, the corresponding coherence length (CL)—which reflects the extent of ordered domain growth—shows an increase from 79.2 Å to 86.3 Å. A similar trend is observed in the π-π stacking direction: While the d-spacing remains unchanged at approximately 3.6 Å, the CL increases from 24.3 to 27.7 Å (tables S9 and S10). These observations indeed suggest that the addition of PQIC not only preserves the intrinsic packing motifs of the host materials but also enhances domain purity and structural coherence. These improvements in crystallinity and molecular ordering facilitate more efficient charge transport and can account for the previously observed enhancements in charge carrier mobilities.

Afterward, the morphology of the active layers was further examined using photoinduced force microscopy (PiFM), a technique that has been widely recognized in recent years for its high spatial resolution in identifying phase distribution and visualizing fibrillization in organic PV blends (*52*–*54*). As shown in Fig. 6 (H to J) and figs. S27 and S28, the PiFM images of the binary (D18:L8-BO), ternary (D18:L8-BO:PQIC), and neat PQIC films reveal distinct morphological features. To differentiate the components, Fourier transform infrared spectroscopy was used for compositional analysis, with characteristic vibrational peaks assigned to D18 (1772 cm^−1^), L8-BO (1422 cm^−1^), and PQIC (1363 cm^−1^), corresponding to green, blue, and red signals in the compositional maps, respectively. Notably, the PQIC film becomes preferentially enriched near the film surface during the drying/film-formation process and displays an intrinsically dense and continuous fibrillar network, which is expected to facilitate efficient and directional charge transport. This favorable morphology is inherited by the D18:L8-BO:PQIC film, in which PQIC forms a well-connected red-colored network with markedly improved continuity, highlighting its beneficial role in enhancing charge percolation pathways.

### Electron-phonon coupling, exciton dynamics, stability evaluation, and large-area device performance

**Fig. 7.**
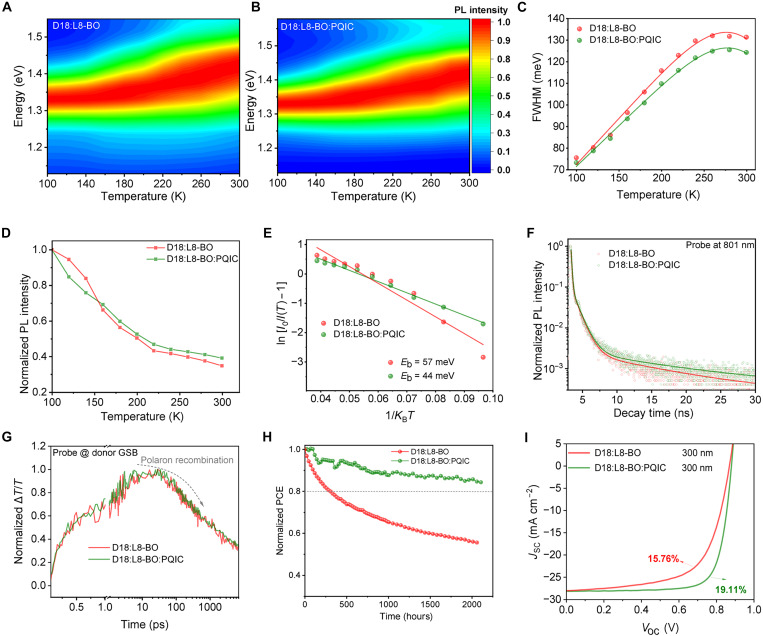
Electron-phonon coupling, exciton dynamics, stability evaluation, and large-area characterization. (**A** and **B**) 2D contour maps of temperature-varied steady-state PL spectra of D18:L8-BO and D18:L8-BO:PQIC films. (**C**) FWHM of PL spectra as a function of temperature. (**D**) Relative PL intensity values alongside temperatures for binary and ternary films. (**E**) The temperature-intensity relationships for assessing binding energy. (**F**) Time-resolved photoluminescence (TRPL) of D18:L8-BO and D18:L8-BO:PQIC films detected at 801 nm. (**G**) Extracted polaron kinetics. (**H**) The normalized PCE roll-off of D18:L8-BO and D18:L8-BO:PQIC devices under maximum power point tracking. (**I**) *J*-*V* characteristics of OSC devices with a 300-nm-thick active layer.

A key characteristic of organic semiconductor materials is their pronounced electron-phonon coupling, which can typically be suppressed using extended fused-ring structures. We therefore conducted temperature-dependent photoluminescence (PL) measurement to investigate this effect (*55*, *56*). According to 2D contour maps of PL spectra in Fig. 7 (A and B), incorporating PQIC can hold distinguishable blueshift emission region compared with D18:L8-BO film. Given the greater overlap in their absorption regions, the Huang–Rhys factor (*S*)—which reflects the degree of electro-phonon coupling—can be qualitatively inferred to be reduced in the ternary film (*57*). Besides, more quantitative and complete information can be gained from appropriate analysis. On the basis of Eq. 1

$$\Gamma \left(T\right)=\Gamma_{i}+\alpha \text{exp}\left(–\frac{E_{a}}{k_{B}T}\right)+\mathrm{bT}$$ (1)

where Γ_i_ denotes the inhomogeneous linewidth of the film’s PL spectra, α is the density of nonradiative recombination center, *E*_a_ means the back charge recombination energy barrier, and *b* is the coefficient of electron-phonon coupling. The FWHM peak values of PL spectra for binary and ternary films are illustrated in Fig. 7C. The fitting results show that the *b* value was 427 μeV K^−1^ for D18:L8-BO binary film, whereas the introduction of PQIC in ternary film markedly reduced the *b* value to 346 μeV K^−1^. Thereby, PQIC with a rigid fused-ring backbone is also proven effective in suppressing electron-phonon interaction.

Meanwhile, the PL intensities of D18:L8-BO and D18:L8-BO:PQIC systems under different temperatures (100 K ~ 300 K) are drawn in Fig. 7D. On the basis of Eq. 2

$$I\left(T\right)=\frac{I_{0}}{1+A\text{exp}\left(-\frac{E_{b}}{K_{B}T}\right)}$$ (2)

where *I*_0_ is the PL intensity at initial temperature, *T* is the temperature, *K*_B_ is the Boltzmann constant, and *E*_b_ means the binding energy; the charge generation barrier *E*_b_ can be rationally assessed (*58*). The fitting graph is visualized by Fig. 7E. As a result, the D18:L8-BO:PQIC film exhibits a substantially lower *E*_b_ of 44 meV, compared to 57 meV for the D18:L8-BO film, suggesting that PQIC can effectively reduce the exciton dissociation barrier and may thereby suppress monomolecular recombination loss.

Besides, temperature-dependent absorption spectra (fig. S29) show that both the D18:L8-BO binary and D18:L8-BO:PQIC ternary systems exhibit a blueshift of the acceptor absorption peak as the temperature increases from 100 to 300 K. Notably, the ternary film displays much smaller temperature-induced variations in the acceptor-related shoulder (~710 nm) and in the donor-dominated absorption band (450 to 650 nm) compared with the binary films. These results indicate that structural disorder may be more effectively suppressed in the ternary system, consistent with the increased CL revealed by GIWAXS. Figure S30 (A to C) presents the time-dependent transient current decays of the binary and ternary devices, which were fitted to derive the corresponding trap-state densities summarized as a function of temperature in fig. S30D. The results indicate that, upon incorporation of PQIC, the average trap-state density of the ternary device decreases from 2.91 × 10^16^ to 2.61 × 10^16^ cm^−3^, thereby effectively suppressing nonradiative recombination pathways. Overall, our findings demonstrate that the rigid fused-ring architecture of PQIC enhances molecular ordering, reduces electron-phonon coupling, and suppresses disorder-induced recombination losses. The charge transfer process was evaluated using time-resolved photoluminescence (TRPL) measurement under 801-nm excitation, as shown in Fig. 7F (*59*, *60*). The exciton lifetime, obtained from double-exponential fitting, is 0.514 ns for the D18:L8-BO film and decreases to 0.402 ns upon the incorporation of PQIC. This result further demonstrates that PQIC facilitates a faster charge transfer process, thereby suppressing geminate recombination and promoting more efficient charge generation.

Furthermore, the interfacial charge dynamics of the two systems were compared using femtosecond transient absorption spectroscopy (fs-TAS) (*61*, *62*). The 2D contour maps of measurement results for two systems are given in fig. S31. Accordingly, the ground state bleaching (GSB) and photoinduced absorption signals can be extracted and analyzed following established protocols. The polaron generation and recombination dynamics are presented in Fig. 7G and fig. S32, which reveal no substantial differences between the D18:L8-BO and D18:L8-BO:PQIC systems. This observation suggests that the incorporation of PQIC does not substantially modify the donor-acceptor interfacial properties. Rather, the optimization of the electronic processes should be attributed to improved phase continuity and the formation of more well-defined pure domains induced by PQIC, which are conducive to more efficient charge transport.

Having established the mechanism by which the PQIC enhances the device efficiency, we further investigated its impact on device stability and thick-film processability, as they are two key factors determining the practical applicability of OSCs. As shown in Fig. 7H, under maximum power point tracking, the D18:L8-BO–based binary device exhibited a *T*_80_ life of only 312 hours. When PQIC was added, the *T*_80_ life of derived devices was substantially improved to more than 2000 hours. These results may originate from the compatibilizing role of PQIC, which may anchor donor and acceptor components, enhances intermolecular interactions, and elevates the glass-transition (*T*_g_) temperature of the active layer (fig. S33), thereby contributing to improved morphological robustness and long-term device stability (*63*). Leveraging the intrinsic dual-channel transport capability of PQIC and the enhanced carrier mobility in ternary systems, we fabricated thick-film devices—an essential step toward OSC commercialization. As shown in Fig. 7I, the ternary devices incorporating PQIC deliver an efficiency of 19.11% at a thickness of 300 nm, retaining 91.8% of the optimal performance. In contrast, the D18:L8-BO binary devices achieve only 15.76% efficiency under the same condition, corresponding to merely 82.3% of the maximum value. These results further highlight the potential of PQIC in improving the processability of thick-film OSCs.

In summary, guided by a fused-ring donor-acceptor integration strategy, we developed a D-A conjugated polymer, PQIC, in which a large fused-ring acceptor unit derived from a high-performance Y-type small-molecule acceptor is covalently incorporated into the backbone of an efficient polymer donor, thereby constructing a rigid and continuous π-conjugated skeleton. This unique architecture endows PQIC with efficient and intrinsically bipolar charge transport, reduced deep trap density, suppressed electron-phonon coupling, and high EL efficiency. Moreover, PQIC serves as an efficient crystallinity modulator and a donor-acceptor compatibilizer, enabling substantial control over molecular packing and morphological stability. When incorporated as a third component into the active layer, PQIC promotes molecular crystallinity, thereby facilitating charge transport, enhancing EL efficiency to suppress nonradiative recombination, and lowering the exciton binding energy (*E*_b_) to accelerate charge transfer. As a result, benefiting from the substantially improved charge management, PQIC-based OSC device can achieve a PCE of 20.81%, with a third-party certified value of 20.60%. In addition, devices incorporating PQIC display thermal stability, robust thick-film, and large-area processability, underscoring its potential for practical application. Overall, this work demonstrates an effective design strategy for conjugated polymers, enabling OSCs with high efficiency, excellent stability, and good processability toward practical applications.

## MATERIALS AND METHODS

### Materials

**BDTF-Sn** was purchased from Solarmer Energy Inc. 3,9-diundecyl-12,13-dihydro-[1,2,5]thiadiazolo[3,4-e]thieno[2″,3″:4′,5′]thieno[2′,3′:4,5]pyrrolo[3,2-g]thieno[2′,3′:4,5]thieno[3,2-b]indole (compound **1**) and benzo[2,1-b:3,4-b′]dithiophene-4,5-dione were purchased from Bide Pharmatech Ltd. Other chemicals and solvents were obtained from commercial sources (Sigma-Aldrich, Acros, Strem, or Alfa Aesar) and used as received.

### Device fabrication and test

The conventional OSCs were fabricated with the structure ITO/2-PACz/Active layer/PDINN/Ag. Indium tin oxide (ITO) substrates were cleaned sequentially by sonication in detergent, deionized water, and isopropanol and then dried in an oven at 60°C overnight. The substrates were treated with oxygen plasma for 5 min, followed by spin coating a 2-PACz solution (0.2 mg/ml) at 4000 rpm for 20 s. The substrates were then annealed at 100°C for 5 min. After washing with ethanol (EtOH) at 3000 rpm once, the blend solution (donor:acceptor = 1:1.3 by weight, with a fixed donor concentration of 3.5 mg/ml) was spin coated onto the treated substrates. In the ternary blend, PQIC was incorporated at a loading of 5 wt % with respect to the donor content in the active layer. The thickness of the active layer was controlled at ~100 nm by adjusting the spin speed. A 5-nm layer of PDINN (0.5 mg/ml in methanol) was subsequently spin coated as the cathode interface. Last, a 90-nm layer of silver was thermally deposited on top of the interface through a shadow mask in a vacuum chamber at a pressure of 1 × 10^−7^ mbar. The device contact area was 0.057 cm^2^, and the illuminated area during testing was 0.04 cm^2^, defined by a mask. The device area used for third-party certification was 0.0354 cm^2^. The *J*-*V* characteristics were measured using a computer-controlled Keithley 2400 source meter under 1-sun illumination from an AM1.5G solar simulator (EnliTech, SS-F5, Taiwan). The light intensity was calibrated using a standard silicon solar cell (certified by the China General Certification Center) to 100 mW/cm^2^ before testing. The EQE spectra were recorded using a QE-R measurement system (EnliTech, QE-R3011, Taiwan).

### General characterizations

PESA measurements were recorded using a KEIKI spectrometer (model AC-3). IPES measurement was performed using a customized Ulvac-Phi LEIPS instrument with Bremsstrahlung isochromatic mode. The ITO samples coated with ~50-nm films of PQIC were used for IPES measurements. UV-Vis absorption spectra were recorded using a SHIMADZU UV-3600 spectrophotometer with corrections for quartz absorption. 2D-GIWAXS measurements were carried out on an Xeuss 3.0 UHR SAXS/WAXS system (Xenocs, France) with a Eiger2 R 1M 2D detector featuring 0.075 mm–by–0.075 mm active pixels in integration mode. The detector was positioned 100/2000 mm downstream from the sample, and the precise sample-to-detector distance was calibrated using a silver behenate standard. The Cu incident x-ray (8 keV) with a 0.9 mm–by–0.9 mm or 0.5 mm–by–0.5 mm spot provided sufficient *q*-space. PiFM results were acquired using a VistaScope microscope from Molecular Vista Inc. PiFM experiments were excited by a pulsed quantum cascade laser (Block Engineering) with a gap-free narrowband tunable wave number of 760 to 1950 cm^−1^. Transient absorption measurement was conducted on the commercial pump-probe femtosecond transient absorption spectrometer HELIOS (Ultrafast System, USA). The capacitance-frequency (*C*-ω) curves were obtained using a Keysight impedance analyzer (E4990A) under dark conditions, spanning a frequency range from 20 to 10^7^ Hz. A dc voltage of 0 V and an ac voltage of 20 mV were applied. This ac voltage was carefully selected to be sufficiently small to minimize data distortion while being large enough to reduce noise interference, ensuring accurate and reliable DOS characterization.
