# Supplementary material for: A donor-acceptor integrated polymer for efficient organic solar cells
Source: Sci Adv. 2026 Apr 8;12(15):eaec1922. doi: 10.1126/sciadv.aec1922 (PMC13060605; doi:10.1126/sciadv.aec1922)
Supplement: Supplementary file 1 — Supplementary Text Figs. S1 to S33 Tables S1 to S10 [file sciadv.aec1922_sm.pdf]

Science Advances
AAAS

Supplementary Materials for

A donor-acceptor integrated polymer for efficient organic solar cells

Lunbi Wu *et al.*

Corresponding author: Sha Liu, shaliu@gbu.edu.cn; Ruijie Ma, ruijiema@buaa.edu.cn; Tao Jia, tjia@gpnu.edu.cn

Sci. Adv. **12**, eaec1922 (2026)
DOI: 10.1126/sciadv.aec1922

This PDF file includes:

Supplementary Text
Figs. S1 to S33
Tables S1 to S10

Supplementary Text

Materials

12,13-Bis(2-decyltetradecyl)-3,9-diundecyl-12,13-dihydro-[1,2,5]thiadiazolo[3,4-e]thieno[2'',3'':4',5']thieno[2',3':4,5]pyrrolo[3,2-g]thieno[2',3':4,5]thieno[3,2-b]indole (**Compound 2**), 11,12-bis(2-decyltetradecyl)-3,8-diundecyl-11,12-dihydrothieno[2'',3'':4',5']thieno[2',3':4,5]pyrrolo[3,2-g]thieno[2',3':4,5]thieno[3,2-b]indole-5,6-diamine (**Compound 3**), dispiro[[1,3]dioxolane-2,4'-benzo[2,1-b:3,4-b']dithiophene-5',2''-[1,3]dioxolane] (**Compound 4**), 2',7'-dibromodispiro[[1,3]dioxolane-2,4'-benzo[2,1-b:3,4-b']dithiophene-5',2''-[1,3]dioxolane] (**Compound 5**), 2',7'-bis(4-(2-butyloctyl)thiophen-2-yl)dispiro[[1,3]dioxolane-2,4'-benzo[2,1-b:3,4-b']dithiophene-5',2''-[1,3]dioxolane] (**Compound 6**), and 2',7'-bis(5-bromo-4-(2-butyloctyl)thiophen-2-yl)dispiro[[1,3]dioxolane-2,4'-benzo[2,1-b:3,4-b']dithiophene-5',2''-[1,3]dioxolane] (**Compound 7**) was synthesized as previously reported.(*1*)

Materials synthesis

*2,7-bis(5-bromo-4-(2-butyloctyl)thiophen-2-yl)benzo[2,1-b:3,4-b']dithiophene-4,5-dione (**Compound 8**)*

2',7'-Bis(5-bromo-4-(2-butyloctyl)thiophen-2-yl)dispiro[[1,3]dioxolane-2,4'-benzo[2,1-b:3,4-b']dithiophene-5',2''-[1,3]dioxolane] (**Compound 7**) (1934 mg, 2 mmol), and fluoroboric acid (aq.) (30mL) were added into flask under N_2 atmosphere. The mixture was stirred at room temperature for 2 hours. After this, the mixture was poured into 200 mL MeOH, and the filter cake was further column chromatography to obtain **Compound 8** as blue solid (1441 mg, yield 82%). ^{1}H NMR (400 MHz, $CDCl_3$) δ 7.36 (s, 2H), 6.86 (s, 2H), 2.47 (d, J = 7.0 Hz, 4H), 1.66 (m, 2H), 1.28 (d, J = 6.5 Hz, 34H), 0.94 – 0.81 (m, 12H). ^{13}C NMR (101 MHz, $CDCl_3$) δ 173.91, 142.83, 141.27, 137.43, 135.78, 134.02, 126.64, 122.68, 111.00, 38.54, 34.19, 33.30, 33.02, 31.90, 29.68, 28.75, 26.49, 23.05, 22.70, 14.15. MASS m/z calcd. for $C_{42}H_{54}Br_2O_2S_4$, 878.940; found, 878.138.

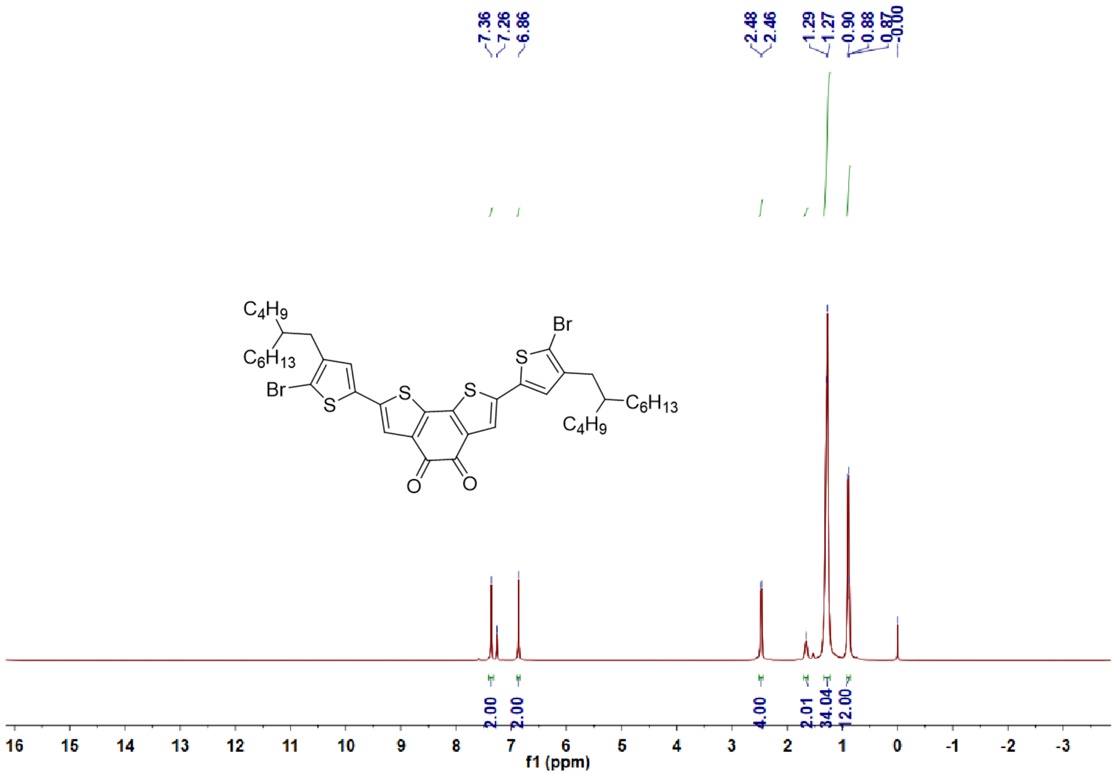

Fig. S1. ^{1}H NMR spectrum of **Compound 8** in $CDCl_3$.

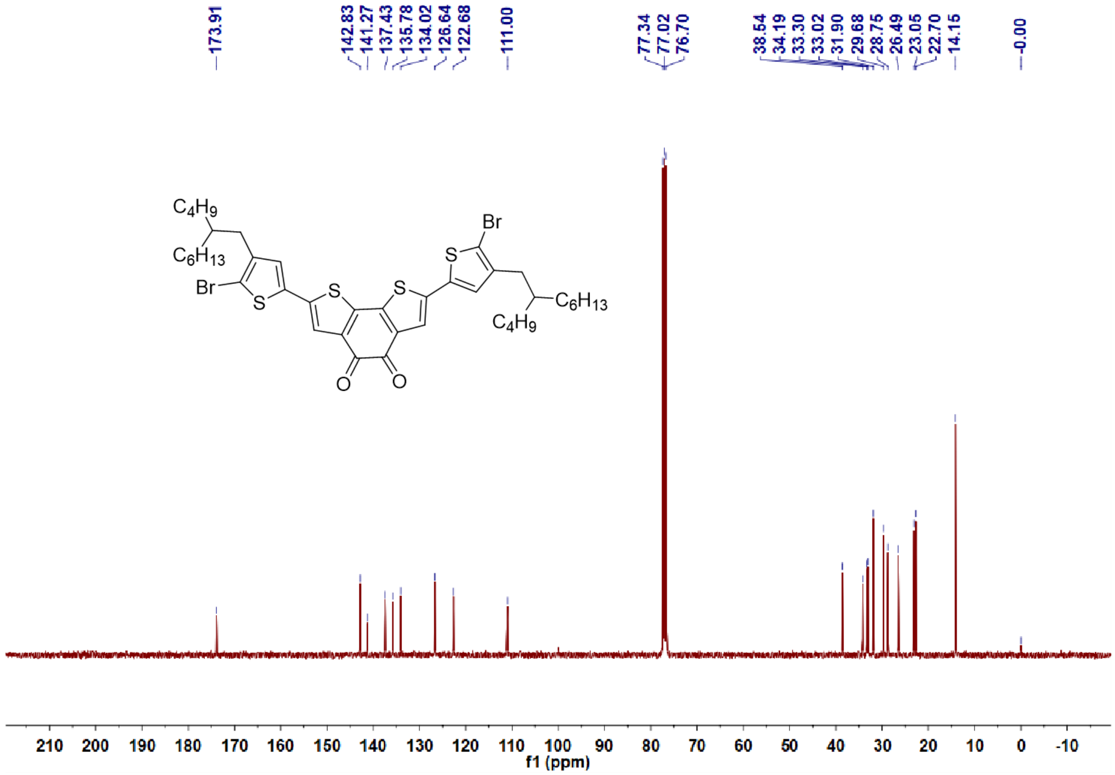

Fig. S2. ^{13}H NMR spectrum of **Compound 8** in $CDCl_3$.

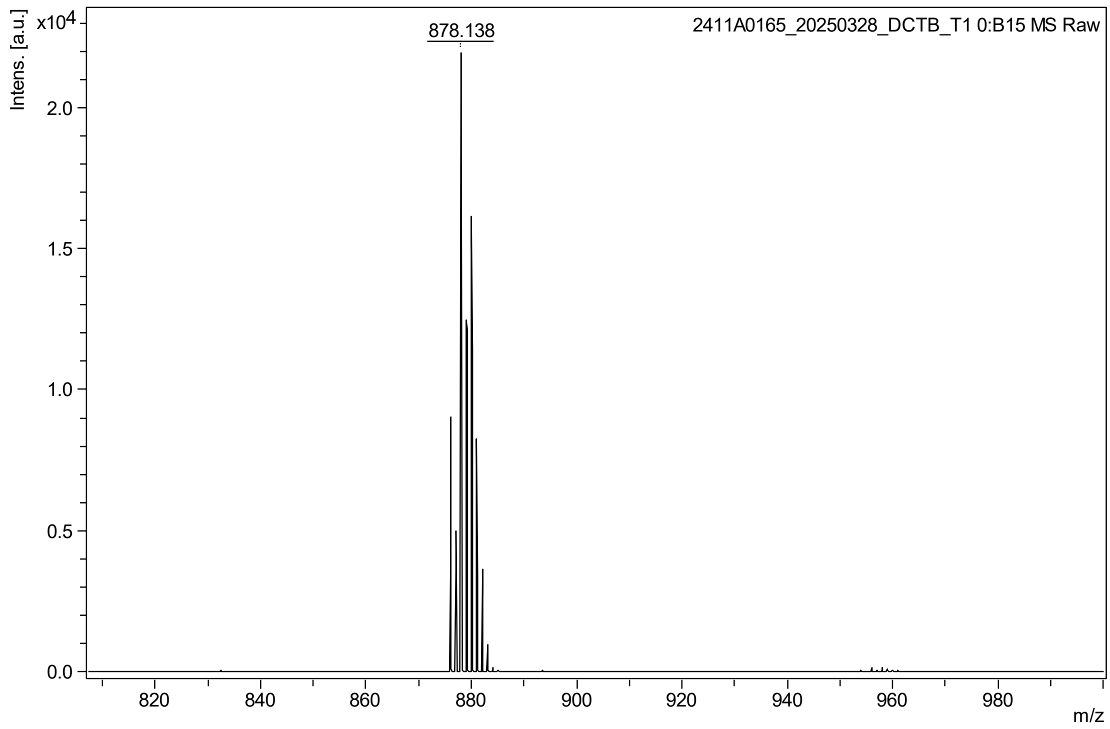

Fig. S3. The mass spectrum of **Compound 8**.

2,17-Bis(5-bromo-4-(2-butyloctyl)thiophen-2-yl)-9,10-bis(2-decyltetradecyl)-6,13-diundecyl-3b,9,10,15a-tetrahydrothieno[2'',3'':4',5']thieno[2',3':4,5]pyrrolo[3,2-a]thieno[2'',3'':4',5']thieno[2',3':4,5]pyrrolo[2,3-c]dithieno[3,2-h:2',3'-j]phenazine ***(Q-H)***

11,12-Bis(2-decyltetradecyl)-3,8-diundecyl-11,12-dihydrothieno[2'',3'':4',5']thieno[2',3':4,5]pyrrolo[3,2-g]thieno[2',3':4,5]thieno[3,2-b]indole-5,6-diamine (**Compound 3**) (1392 mg, 1 mmol) was added into a flask containing 30 mL EtOH under N_2 atmosphere. **Compound 8** (878.9 mg, 1 mmol) was then added in three portions. The reaction mixture was stirred at 100 °C for 2 hours. After cooling to room temperature, the reaction mixture was poured into water (300 mL) and extracted with dichloromethane (DCM). The organic layer was collected, and the solvent was removed under reduced pressure. The resulting residue was purified by column chromatography on silica gel using a petroleum ether/DCM mixture as the eluent, affording Q-H as a red solid. (1900 mg, yield 85%). ^{1}H NMR (400 MHz, $CDCl_3$) δ 8.63 (s, 2H), 7.09 (s, 2H), 7.04 (s, 2H), 4.70 (d, J = 7.3 Hz, 4H), 2.95 (t, J = 7.5

Hz,4H), 2.57 (d, $J = 7.0$ Hz,4H), 2.18 (s, 2H), 1.97 (dt, $J = 15.0$, 7.6 Hz, 4H), 1.77 (s, 2H), 1.41 – 1.22 (m, 80H), 1.02 – 0.73 (m, 94H). ^{13}C NMR (101 MHz, $CDCl_3$) δ 143.30, 142.38, 137.18, 137.01, 136.71, 136.47, 136.34, 135.88, 135.18, 131.95, 131.27, 126.01, 123.51, 123.10, 120.67, 118.65, 117.58, 109.49, 55.19, 38.77, 38.56, 34.32, 33.43, 33.09, 31.97, 31.92, 30.58, 29.76, 29.68, 29.62, 29.53, 29.44, 29.41, 29.34, 28.90, 28.82, 26.59, 25.66, 23.11, 22.72, 14.13. MALDI-TOF m/z calcd. for $C_{130}H_{200}Br_2N_4S_8$, 2235.346; found, 2235.190.

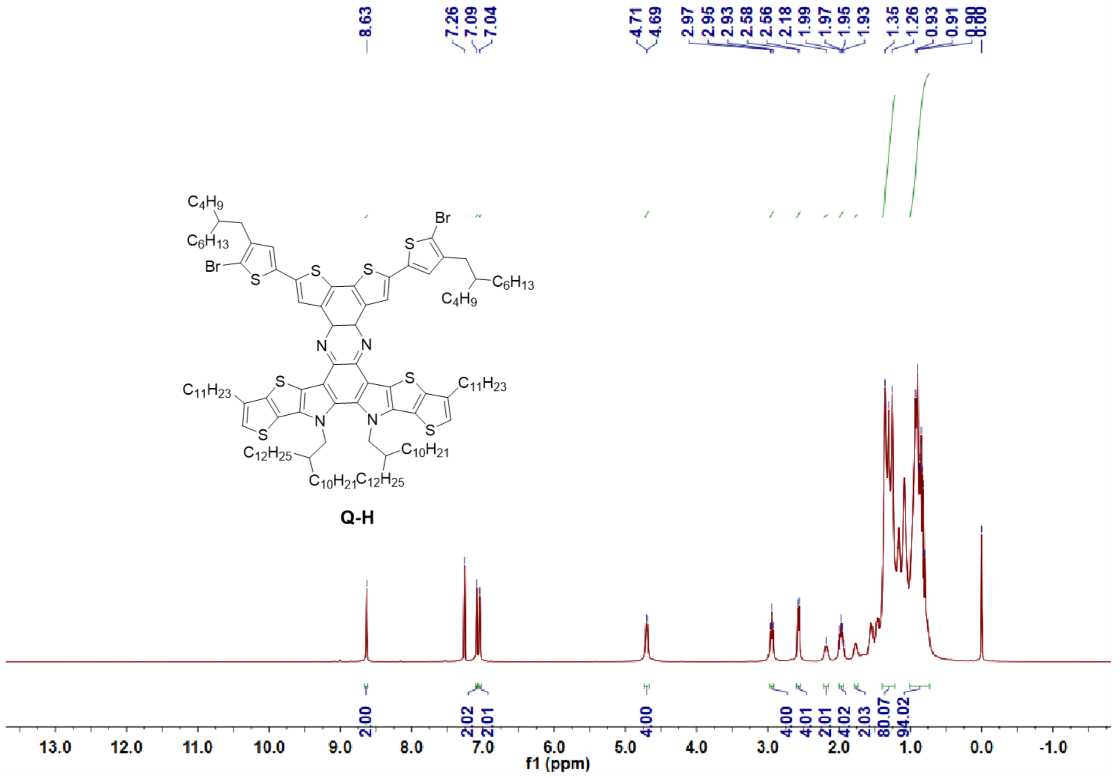

Fig. S4. ^{1}H NMR spectrum of **Q-H** in $CDCl_3$.

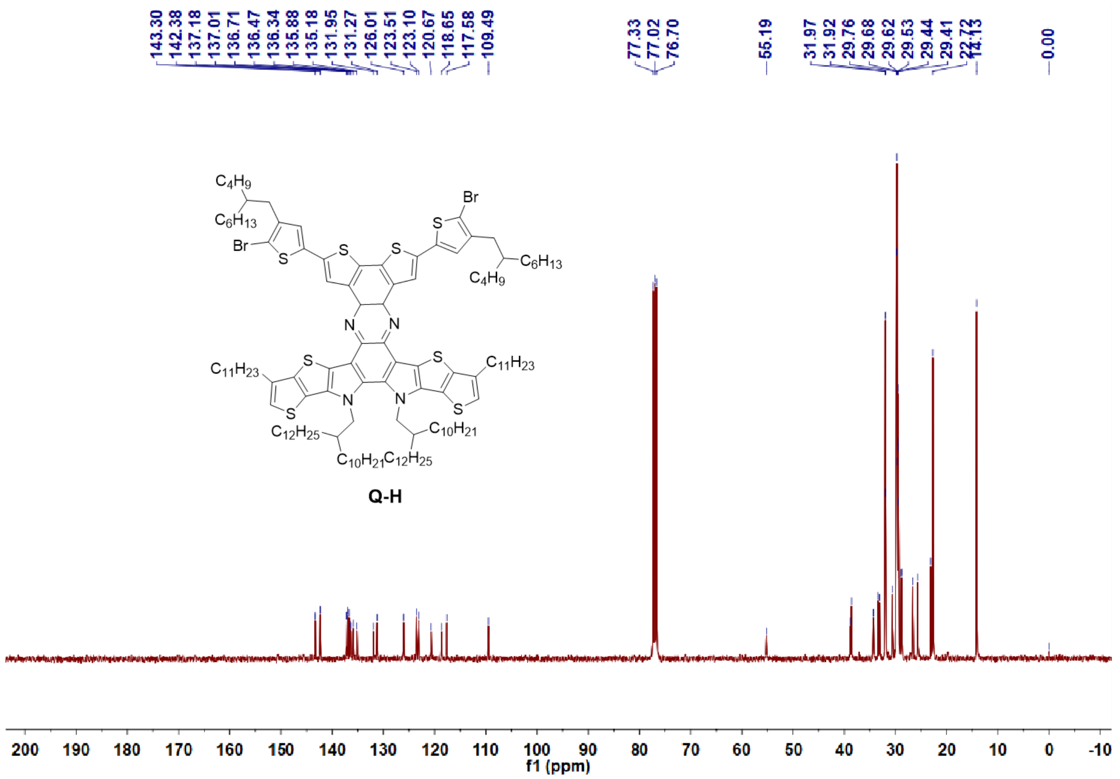

Fig. S5. ^{13}C NMR spectrum of **Q-H** in $CDCl_3$.

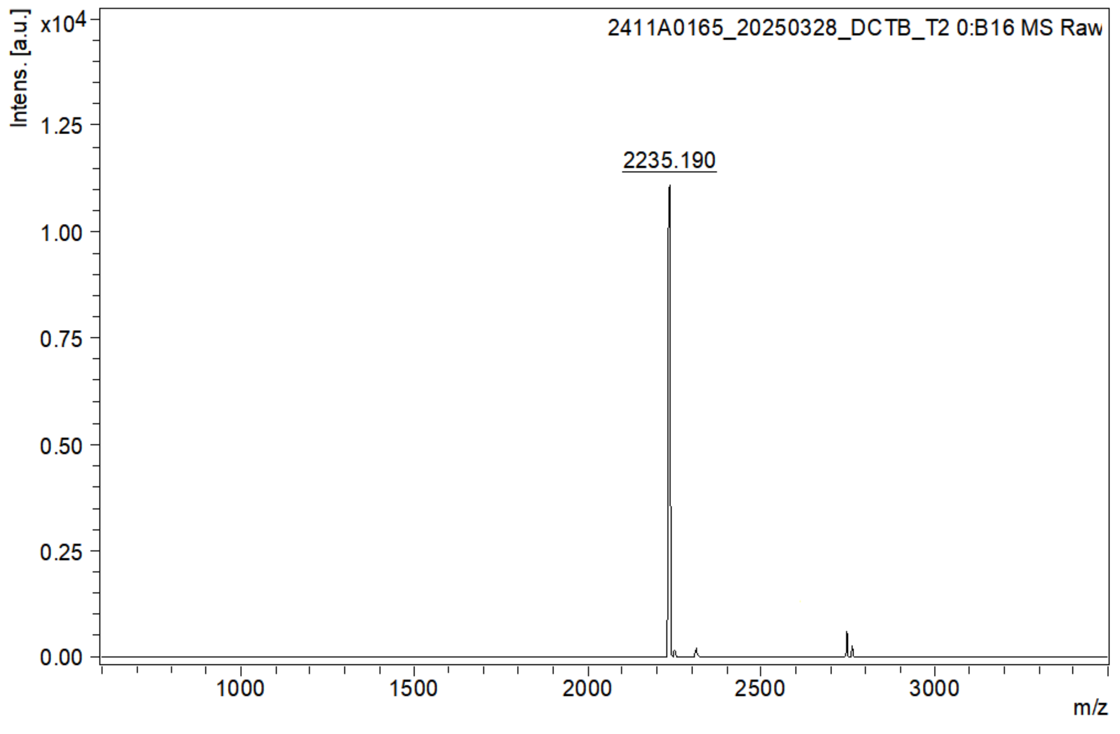

Fig. S6. The mass spectrum of **Q-H**.

2,17-bis(5-bromo-4-(2-butyloctyl)thiophen-2-yl)-9,10-bis(2-decyltetradecyl)-6,13-diundecyl-9,10-dihydrothieno[2'',3'':4',5']thieno[2',3':4,5]pyrrolo[3,2-

a]thieno[2'',3'':4',5']thieno[2',3':4,5]pyrrolo[2,3-c]dithieno[3,2-h:2',3'-j]phenazine-7,12-dicarbaldehyde (Q-CHO)

To a two-neck flask containing dried DMF (50 mL), $POCl_3$ (1 mL) was slowly added under argon atmosphere. After stirring at room temperature for 30 min, a solution of **Q-H** (1118 mg, 0.5 mmol) in dried DCM (10 mL) was added to the flask. The reaction mixture was then stirred at 80 ºC for 3 hours. After cooling to room temperature, the reaction mixture was slowly poured into ice saturated Na_2CO_3 solution (300 mL). The product was extracted with dichloromethane (DCM). After evaporating the organic solvent by reduced pressure, the residue was purified by column chromatography on silica gel using DCM as eluent to afford **Q-CHO** as red solid (1020 mg, yield 89%). ^{1}H NMR (400 MHz, $CDCl_3$) δ 10.20 (s, 2H), 8.48 (d, J = 3.0 Hz, 2H), 6.92 (d, J = 4.2 Hz, 4H), 4.76 (d, J = 6.9 Hz, 4H), 3.31 (t, J = 7.3 Hz, 4H), 2.50 (d, J = 6.6 Hz, 4H), 2.21 (m, 2H), 2.10 – 1.99 (m, 4H), 1.72 (s, 2H), 1.58 (m, 4H), 1.51 – 1.43 (m, 6H), 1.31 (m, 48H), 1.22 (m, 28H), 1.09 (m, 32H), 0.97 (m, 32H), 0.82 (m, 24H). ^{13}C NMR (101 MHz, $CDCl_3$) δ 180.68, 145.99, 143.33, 141.50, 135.89, 135.75, 135.67, 135.45, 134.89, 134.73, 134.57, 131.29, 128.62, 126.78, 125.02, 119.26, 117.40, 108.70, 54.43, 38.04, 37.58, 33.31, 32.37, 32.04, 30.92, 30.89, 30.86, 29.51, 28.72, 28.70, 28.67, 28.62, 28.55, 28.51, 28.45, 28.34, 28.28, 27.77, 27.23, 25.56, 24.64, 22.07, 21.71, 21.66, 21.64, 13.15, 13.13, 13.09, 13.06. MALDI-TOF m/z calcd. for $C_{132}H_{200}Br_2N_4O_2S_8$, 2291.366; found, 2291.183.

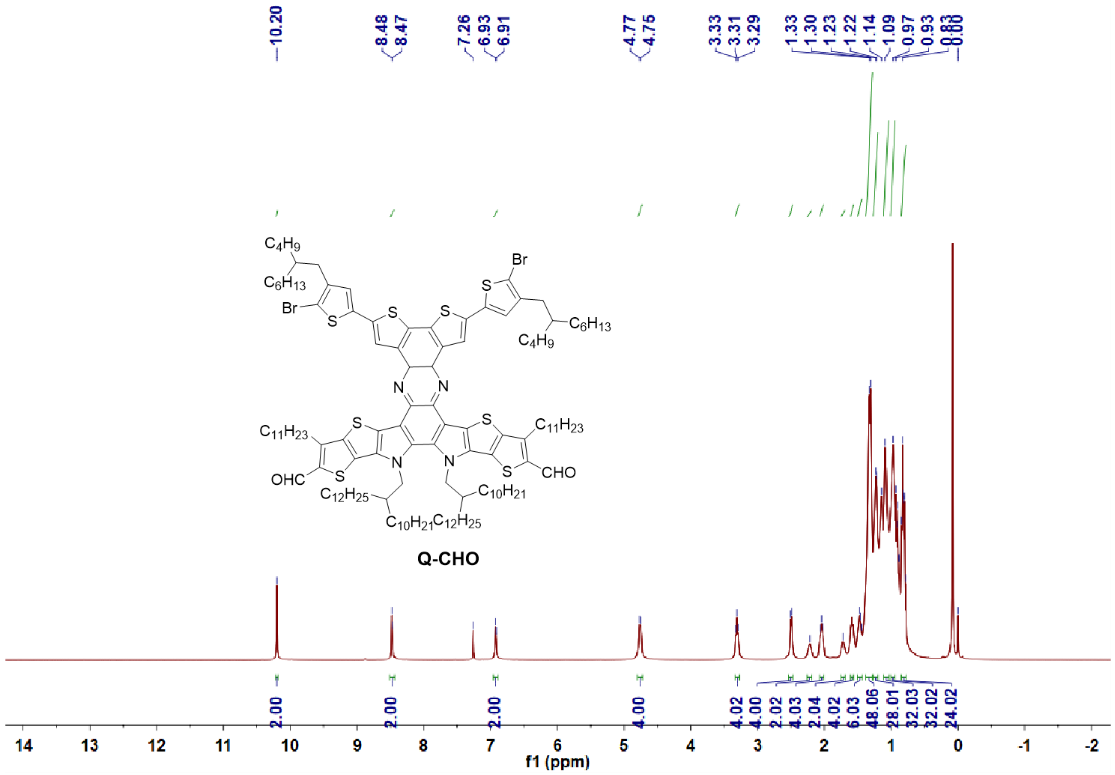

Fig. S7. ^{1}H NMR spectrum of **Q-CHO** in $C_2D_2Cl_4$ at 100 ºC..

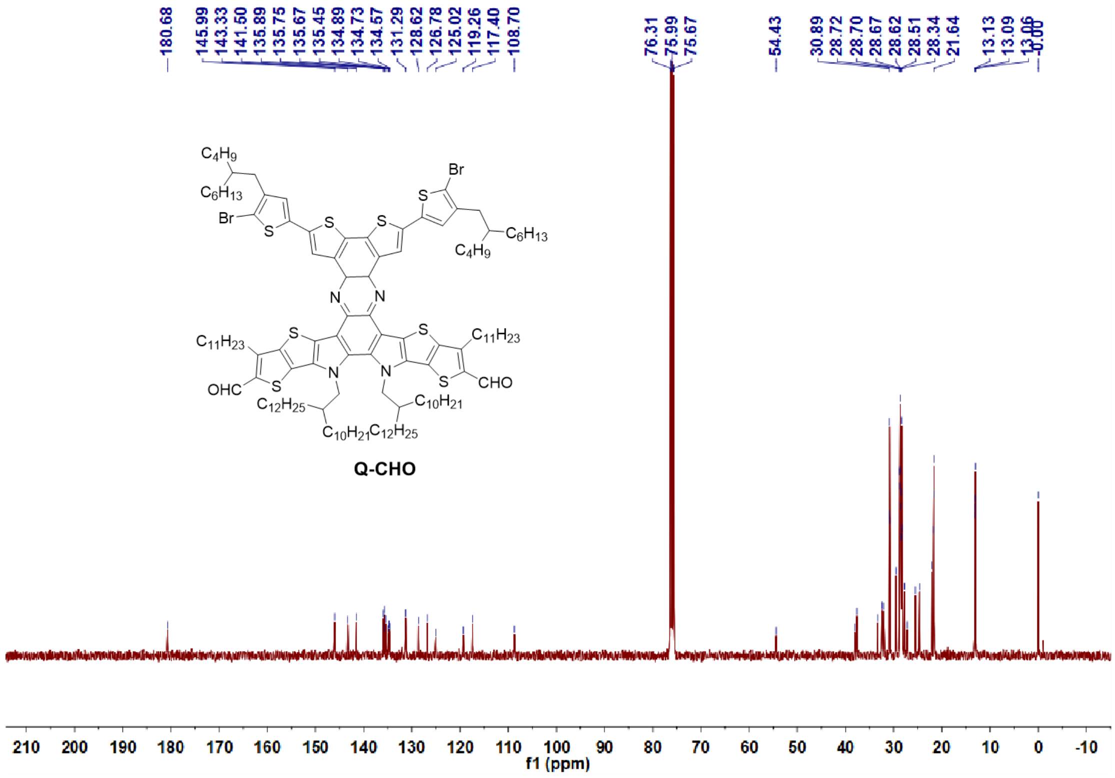

Fig. S8. [13]H NMR spectrum of **Q-CHO** in $C_2D_2Cl_4$ at 100 ºC.

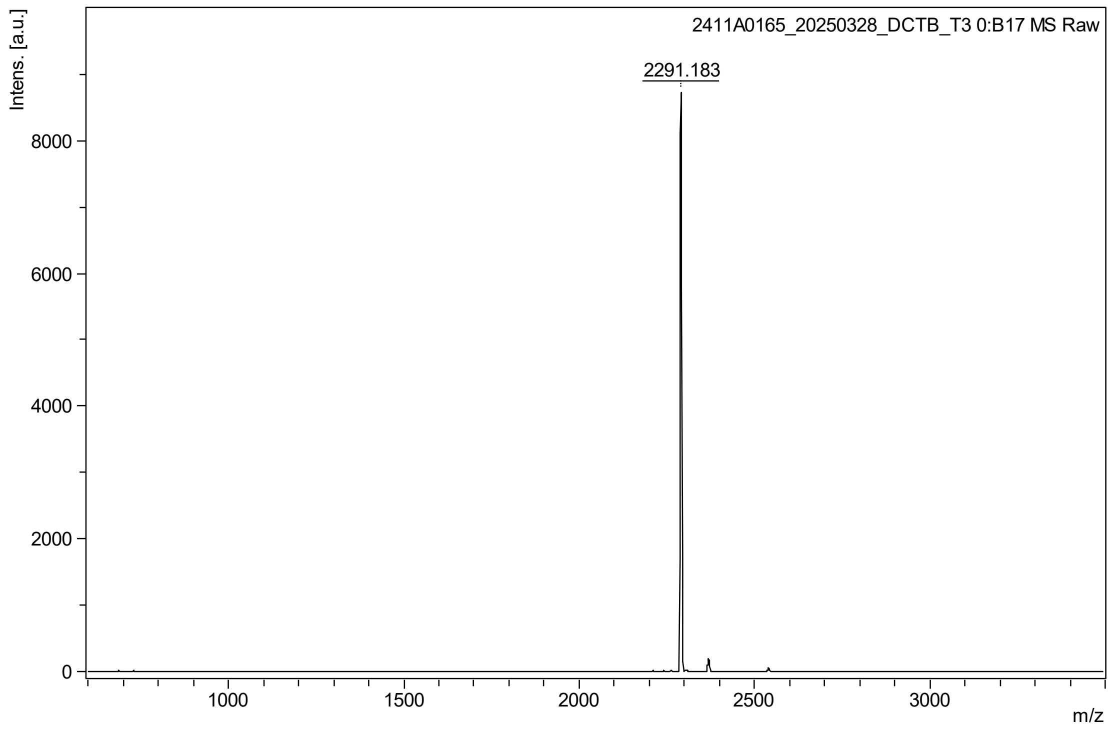

Fig. S9. The mass spectrum of **Q-CHO**.

Q-IC:

To a two-neck flask containing Q-CHO (917 mg, 0.4 mmol) and the isomeric mixed end groups of 2-(5-chloro-3-oxo-2,3-dihydro-1H-inden-1-ylidene)malononitrile and 2-(6-chloro-3-oxo-2,3-dihydro-1H-inden-1-ylidene)malononitrile (IC-Cl) (912 mg, 4 mmol) were added into a flask containing 30 mL chloroform. Then pyridine (0.5 mL) was added and the reaction mixture was stirred at 60 ºC for 18 hours. The residue was purified by column chromatography using PE/DCM (*v*/*v*, 1:1) to give the target product **Q-IC** as a dark solid (955 mg, yield 88%). ^{1}H NMR (400 MHz, $CDCl_3$) δ 9.04 (m, 2H), 8.40 (s, 2H), 8.30 (dd, *J* = 21.3, 9.6 Hz, 2H), 7.74 (d, *J* = 14.9 Hz, 2H), 7.57 (dd, *J* = 21.5, 7.9 Hz, 2H), 6.95 (s, 2H), 4.94 (m, 4H), 3.20 (m, 4H), 2.64 – 2.33 (m, 6H), 1.89 (m, 4H), 1.74 (m, 2H), 1.32 (m, 48H), 1.11 (m, 96H), 0.92 (m, 12H), 0.82 – 0.72 (m, 18H). ^{13}C NMR (126 MHz, $C_2D_2Cl_4$) δ 186.75, 142.95, 141.59, 141.01, 138.75, 138.32, 137.45, 136.51, 136.39, 136.19, 135.42, 134.59, 134.12, 133.37, 131.82, 126.81, 126.29, 125.26, 124.47, 123.69, 120.56, 115.30, 115.06, 114.79, 110.44, 56.46, 39.77, 38.79, 34.82, 33.98, 33.61, 31.95, 31.44, 31.10, 30.05, 29.92, 29.73, 29.68, 29.64, 29.60,

29.49, 29.46, 29.36, 29.33, 29.30, 29.09, 26.83, 26.26, 23.11, 22.72, 22.65, 14.09, 14.01.

MALDI-TOF m/z calcd. for $C_{156}H_{206}Br_2Cl_2N_8O_2S_8$, 2712.606; found, 2712.181.

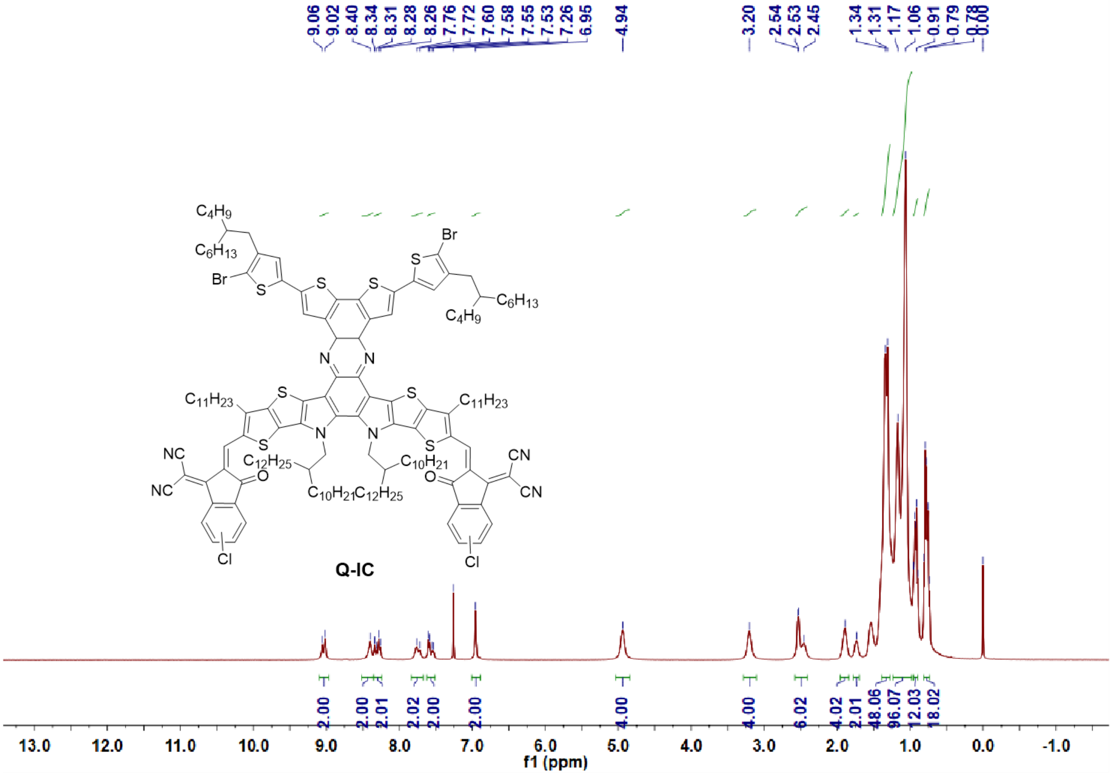

Fig. S10. ^{1}H NMR spectrum of **Q-IC** in $C_2D_2Cl_4$ at 100 °C..

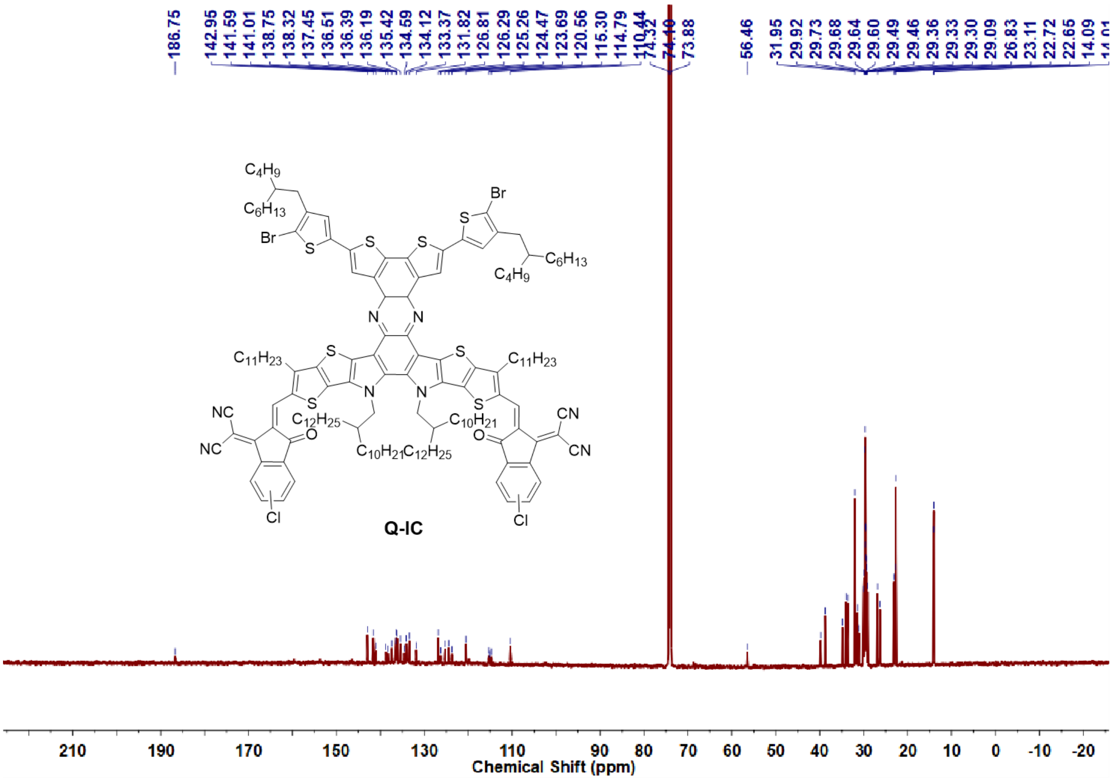

Fig. S11. ^{13}H NMR spectrum of **Q-IC** in $C_2D_2Cl_4$ at 100 °C.

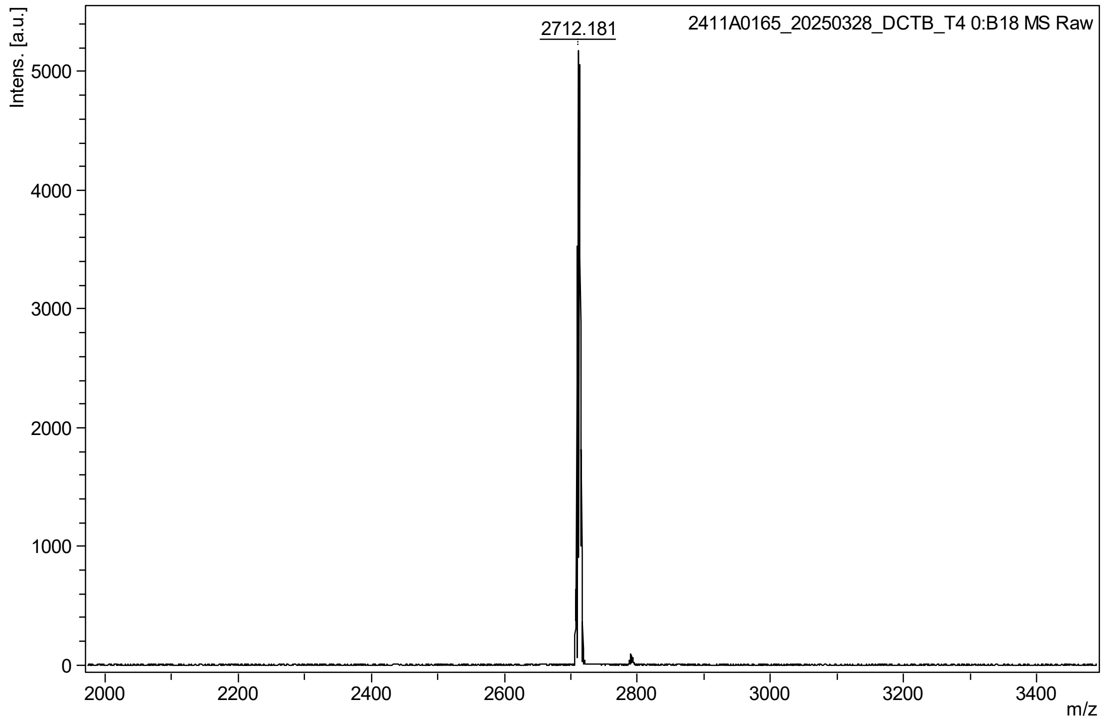

Fig. S12. The mass spectrum of **Q-IC**.

PQIC

Monomer **BDTF-DSn** (94.05 mg, 0.15 mmol), **Q-IC** (271.2 mg, 0.1 mmol), $Pd_2(dba)_3$ (3mg) and P(*o*-tol)$_3$ (4.5 mg) were combined in a 48 mL sealed tube. Dry chlorobenzene (CB) (10 mL) was added under argon atmosphere. The mixture was reacted at 110 °C for 72 h. After cooled down to room temperature, the reactant mixture was poured into MeOH (300 mL). The precipitate was filtered and Soxhlet extracted with methanol, hexane, dichloromethane and chloroform. The ingredient extracted from chloroform was concentrated and precipitated into 500 mL methanol, filtered and dried under vacuum to give the black filament (253 mg, yield 80 %, M_n = 31.1 kDa, M_w = 57.5 kDa); ^{1}H NMR (500 MHz, $C_2D_2Cl_4$) δ 9.25 (s, 2H), 8.82 – 8.56 (m, 4H), 7.84 (dd, J = 22.4, 14.5 Hz, 4H), 7.68 (d, J = 7.6 Hz, 2H), 7.41 (s, 2H), 7.30 (s, 2H), 4.92 (m, 2H), 3.40 (m, 4H), 3.06 – 2.78 (m, 8H), 2.27 (m, 2H), 2.08 (m, 4H), 1.98 – 1.85 (m, 2H), 1.86 – 1.73 (m, 2H), 1.69 – 1.57 (m, 4H), 1.57 – 1.26 (m, 64H), 1.26 – 0.98 (m, 100H), 0.91 (m, 18H), 0.86 – 0.74 (m, 18H).

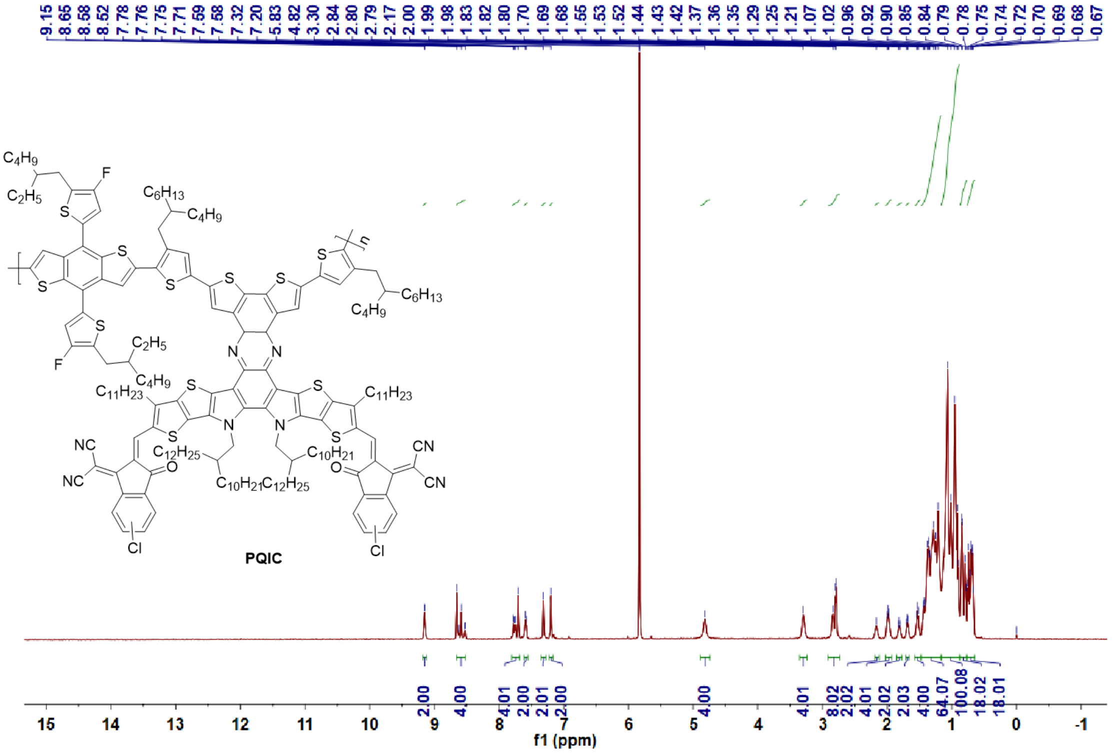

Fig. S13. The high-temperature ^{1}H NMR spectrum of PQIC in $C_2D_2Cl_4$ at 135 ºC.

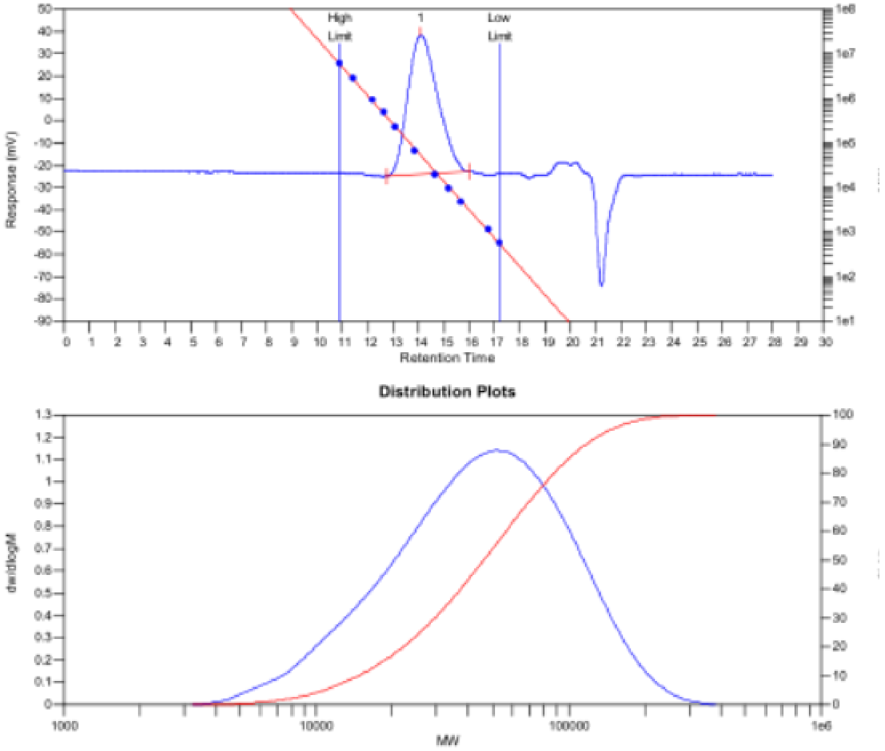

MW Averages

Peak No	Mp	Mn	Mw	Mz	Mz+1	Mv	PD
1	52311	31079	57457	91221	126367	52610	1.84874

Processed Peaks

Peak No	Name	Start RT (mins)	Max RT (mins)	End RT (mins)	Pk Height (mV)	% Height	Area (mV.secs)	% Area
1		12.75	14.10	16.00	62.3931	100	5145.91	100

Fig. S14. The high-temperature GPC of PQIC in trichlorobenzene at 150 °C.

UV−Vis Absorption Spectra

For the solution absorption measurements, the materials were dissolved in chloroform (CF) at a concentration of approximately 1 × 10^{-5}mol/L. Thin solid films (~100 nm thick) for spectral analysis were prepared by spin-coating the CF solutions onto quartz substrates.

PESA Measurements

The power setting of 30 nW and a power number of 0.5 were used for PESA measurement. Samples for PESA measurement were 50 nm prepared on the glass substrate.

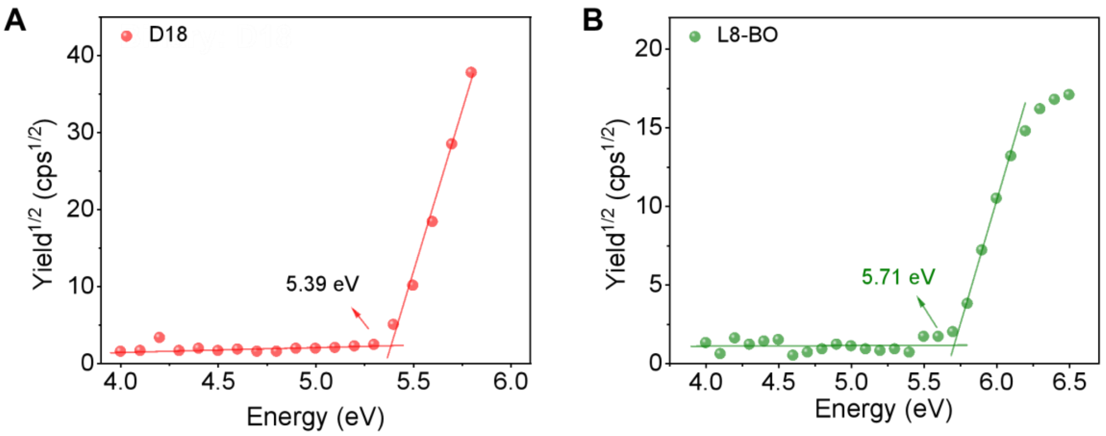

Fig. S15. The derived HOMO energy levels by PESA measurements of (**A**) D18 and (**B**) L8-BO.

Theoretical Calculations

Gaussian 16 (Revision C.02) code(*2*) was used for density functional theory (DFT) calculations, including structure optimization and single-point energy calculations. For structure optimization, the B3LYP-D3(BJ)/Def2SVP (*3-5*) level of theory was applied to all compounds, in order to balance accuracy with reduced computational cost and time. The Wavefunction software Multiwfn(*6*) and VMD (*7*) were used to analyze the electrostatic potential (ESP) and the Noncovalent Interaction (NCI). These analyses of ESP are commonly performed on molecular van der Waals (vdW) surfaces. The

noncovalent interaction (NCI) method, which is also known as reduced density gradient (RDG) method, is used to study weak interaction.(*8*) The definition of the RDG function is shown below, it is essentially a dimensionless form of electron density gradient norm function:

$$RDG(r) = \frac{1}{2(3\pi^2)^{1/3}} \frac{|\nabla\rho(\boldsymbol{r})|}{\rho(\boldsymbol{r})^{4/3}}$$

For remaining regions ("Around chemical bond" and " Weak interaction region"), only weak interaction regions were revealed

Device Performance of Single PQIC-based OSCs

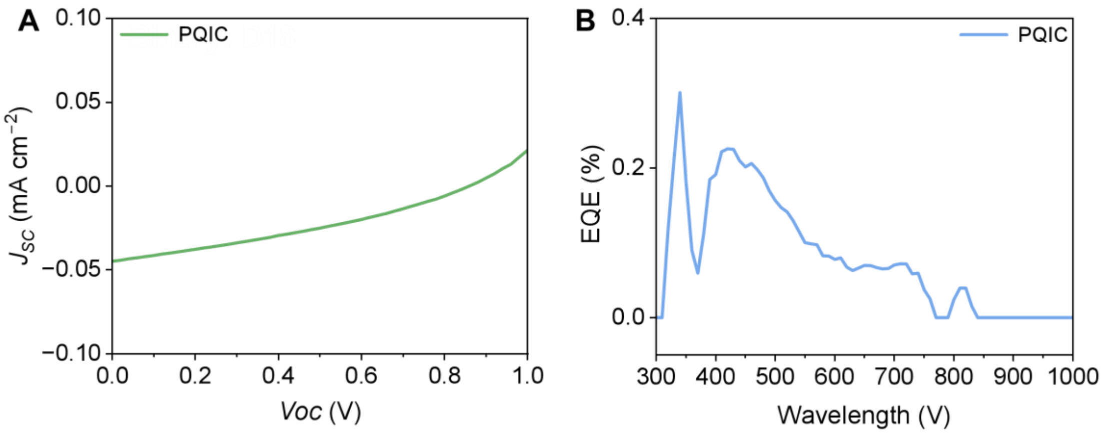

Fig. S16. (**A**) *J-V* characteristics of a single-component PQIC-based OSCs. (**B**) EQE spectra.

Third-party Certified Report

华南国家计量测试中心
广东省计量科学研究院
SOUTH CHINA NATIONAL CENTER OF METROLOGY
GUANGDONG INSTITUTE OF METROLOGY

校 准 证 书
CALIBRATION CERTIFICATE

证书编号 Certificate No. NYX202500437　　　　第 1 页，共 5 页 Page of

客户名称 Name of the Customer	广东技术师范大学 Guangdong Polytechnic Normal University
联络信息 Contact Information	广东省广州市天河区中山大道239号 No.?293 Zhongshan Avenue, Tianhe District, Guangzhou, Guangdong Province
计量器具名称 Description	非富勒烯有机太阳电池 Non-fullerene organic solar cell
型号/规格 Model/Type	非富勒烯有机太阳电池 Non-fullerene organic solar cell
制造厂 Manufacturer	广东技术师范大学 Guangdong Polytechnic Normal University
出厂编号 Serial No.	W-7
设备管理编号 Equipment No.	----
接收日期 Receipt on	2025 年 Y 07 月 M 22 日 D
结论 Conclusion	见校准结果 Shown in the results of calibration
校准日期 Calibration on	2025 年 Y 07 月 M 22 日 D
发布日期 Issue on	2025 年 Y 07 月 M 28 日 D

批 准 Authorized by 吴江宏
核 验 Reviewed by 林鼎添
校 准 Calibrated by 梅书刚

华南国家计量测试中心 证书专用章 Stamp

扫一扫查真伪

实验室地址：广东省东莞市石排镇东园大道石排段152号　邮政编码：523343
电话：(8620)86594172 传真：(8620)86590743 投诉电话：(8620)36611242 E-mail：scm@scm.com.cn
Add: No.1,Miaobianwang Section, Dongyuan Road South, Shipai Town, Dongguan, Guangdong
Post Code: 523343 Tel: (8620)86594172 Fax: (8620)86590743 Complaint Tel: (8620)36611242
证书真伪查询：www.scm.com.cn；cert.scm.com.cn Certificate AuthenticityIdentify: www.scm.com.cn；cert.scm.com.cn

华南国家计量测试中心
广东省计量科学研究院
SOUTH CHINA NATIONAL CENTER OF METROLOGY
GUANGDONG INSTITUTE OF METROLOGY

说　明
DIRECTIONS

证书编号 Certificate No. NYX202500437　　　　第 2 页，共 5 页 Page of

1. 本中心是国家市场监督管理总局在华南地区设立的国家法定计量检定机构，本中心的质量管理体系符合ISO/IEC 17025:2017标准的要求。
   This laboratory is the National Legal Metrological Verification Institution in southern China set up by the State Administration for Market Regulation. The quality system is in accordance with ISO/IEC 17025:2017.
2. 本中心所出具的数据均可溯源至国家计量基准和/或国际单位制(SI)。
   All data issued by this laboratory are traceable to national primary standards and/or International System of Units (SI).
3. 校准地点、环境条件：
   Location and environmental conditions of the calibration:
   地点 Place 本院二基地A1-402　温度 Temperature (25±2) ℃　相对湿度 R.H. (50±5) %
4. 本次校准的技术依据：
   Reference documents for the calibration:
   JJF1622-2017 太阳电池校准规范：光电性能　C.S.for Solar Cells: Photoelectric Properties
5. 本次校准所使用的主要计量标准器具：
   Major standards of measurement used in the calibration:

设备名称/型号规格/测量范围 Name of Equipment /Model/Type/Range	编号 Serial No.	证书号/有效期/溯源单位 Certificate No./Due Date /Traceability to	计量特性 Metrological Characteristic
ABET 稳态太阳模拟器 1000W full spectrum solar simulator /SUN3000/(300～1300)W/m²	374	NYX202400537 /2025-12-03 /本中心	光谱匹配度：A级 辐照度不均匀性：A级 辐照度不稳性：A级
标准太阳能电池 Standard Solar Cell /RR_257_0/ Isc：(1～200)m A	13/01/2014	GXgf2024-06295 /2025-09-24 /国家计量院	U_{rel}=2.2%， k=2
标准源表 Standard Source Meter /2420/(0～60)V,(0～3)A	4051271	DBB202503306 /2026-02-13 /本中心	电压:U_{rel}=0.1%,电流:U_{rel}=0.1%(k=2) DCV:U_{rel}=0.1%,DCA:U_{rel}=0.1%(k=2)

— 本说明页以下空白 —

注： Note:
1. 本证书校准结果只与受校准仪器有关。 The results relate only to the items calibrated.
2. 未经本机构书面批准，不得部分复制此证书。 This certificate shall not be reproduced except in full, without the written approval of our laboratory.
3. “客户名称”、“联络信息”由委托方提供，“制造厂”、“型号规格”、“出厂编号”以及“设备编号”为仪器上标注，委托方对上面内容如有异议，须在收到证书后二十个工作日内提出。
   The information **Name of the Customer** and **Contact Information** are provided by client, and the **Manufacturer, Model/Type, Serial No.** and **Equipment No.** are marked on the items.Client shall submit any objection within 20 working days after receiving the certificate for the information above.

说　明

DIRECTIONS

证书编号 NYX202500437
Certificate No.

（续5）

设备名称/型号规格/测量范围 Name of Equipment /Model/Type/Range	编号 Serial No.	证书号/有效期/溯源单位 Certificate No./Due Date /Traceability to	计量特性 Metrological Characteristic
读数显微镜 Microscope for Reading /JC-10/(0~6)mm	960129	CYY202403227 /2025-11-26 /本中心	允差:10.0 μm MPE:10.0 μm

— 本说明页以下空白 —

校 准 结 果

RESULTS OF CALIBRATION

证书编号 NYX202500437　　原始记录号 NYX202500437
Certificate No.　　Record No.

1、外观检查：　符合要求
Apparent Inspection:　Pass.

2、测试条件：　温度(25±2)℃；辐照度1000W/m^2。
Test conditions:　Temperature: (25±2)℃；Irradiance：1000W/m^2.

3、电流-电压特性曲线和功率-电压特性曲线：
I-V and P-V curves:

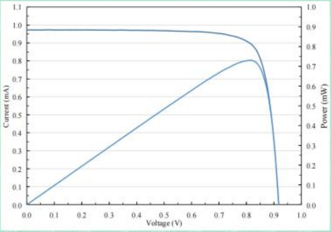

图1 电流-电压特性曲线和功率-电压特性曲线
Figure1 I-V and P-V characteristic curves

4、光电性能参数：
Results of photoelectric properties:

表1(Table 1)

面积 Area	短路电流密度J_{sc} Short ciruit current density	短路电流 I_{sc} Short ciruit current	开路电压 V_{oc} Open circuit voltage	填充因子 FF Fill factor	最大功率 P_m Maximum power	最佳工作电流I_m Optimum working current	最佳工作电压V_m Optimum working voltage	转换效率 η Efficiency
cm^2	mA/cm^2	mA	V	%	mW	mA	V	%
0.0354	27.46	0.972	0.917	81.79	0.729	0.889	0.820	20.60

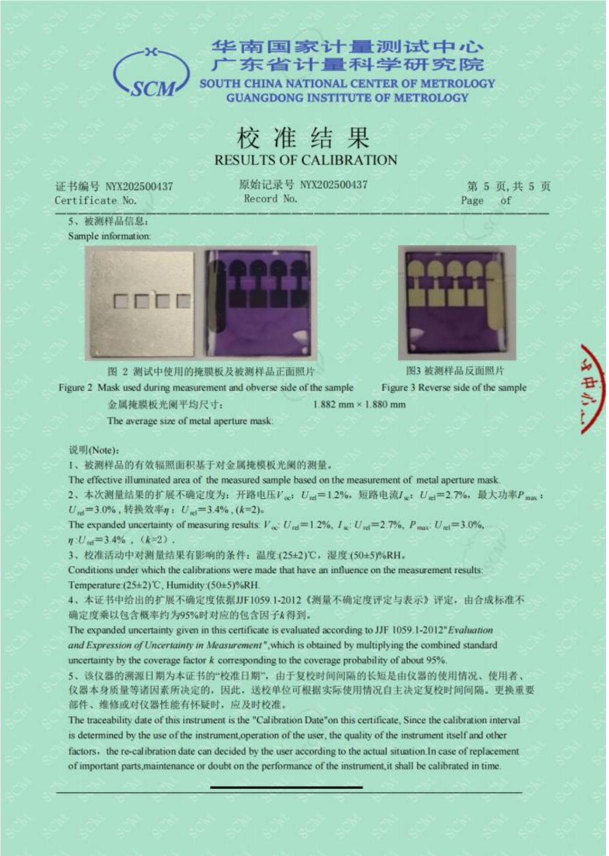
华南国家计量测试中心
广东省计量科学研究院
SOUTH CHINA NATIONAL CENTER OF METROLOGY
GUANGDONG INSTITUTE OF METROLOGY

校 准 结 果
RESULTS OF CALIBRATION

证书编号 NYX202500437 Certificate No. 原始记录号 NYX202500437 Record No. 第 5 页，共 5 页 Page of

5、被测样品信息：
Sample information:

图 2 测试中使用的掩膜板及被测样品正面照片
Figure 2 Mask used during measurement and obverse side of the sample

图3 被测样品反面照片
Figure 3 Reverse side of the sample

金属掩膜板光阑平均尺寸： 1.882 mm × 1.880 mm
The average size of metal aperture mask:

说明(Note)：
1、被测样品的有效辐照面积基于对金属掩模板光阑的测量。
The effective illuminated area of the measured sample based on the measurement of metal aperture mask.
2、本次测量结果的扩展不确定度为：开路电压V_{oc}：U_{rel}=1.2%，短路电流I_{sc}：U_{rel}=2.7%，最大功率P_{max}：U_{rel}=3.0%，转换效率η：U_{rel}=3.4%，(k=2)。
The expanded uncertainty of measuring results: V_{oc}: U_{rel}=1.2%, I_{sc}: U_{rel}=2.7%, P_{max}: U_{rel}=3.0%, η: U_{rel}=3.4%，（k=2）.
3、校准活动中对测量结果有影响的条件：温度:(25±2)℃，湿度:(50±5)%RH。
Conditions under which the calibrations were made that have an influence on the measurement results: Temperature:(25±2)℃, Humidity:(50±5)%RH.
4、本证书中给出的扩展不确定度依据JJF1059.1-2012《测量不确定度评定与表示》评定，由合成标准不确定度乘以包含概率约为95%时对应的包含因子k得到。
The expanded uncertainty given in this certificate is evaluated according to JJF 1059.1-2012"*Evaluation and Expression of Uncertainty in Measurement*",which is obtained by multiplying the combined standard uncertainty by the coverage factor k corresponding to the coverage probability of about 95%.
5、该仪器的溯源日期为本证书的"校准日期"，由于复校时间间隔的长短是由仪器的使用情况、使用者、仪器本身质量等诸因素所决定的，因此，送校单位可根据实际使用情况自主决定复校时间间隔。更换重要部件、维修或对仪器性能有怀疑时，应及时校准。
The traceability date of this instrument is the "Calibration Date"on this certificate, Since the calibration interval is determined by the use of the instrument,operation of the user, the quality of the instrument itself and other factors，the re-calibration date can decided by the user according to the actual situation.In case of replacement of important parts,maintenance or doubt on the performance of the instrument,it shall be calibrated in time.

Fig. S17. The third-party certified report.

Photovoltaic Performances of ternary OSCs

Table S1. Statistical sheet of V_{OC} × FF versus PCE data of bulk-heterojunction OSCs reported in recent year.

Active Layer	J_{SC} [mA cm^{-2}]	V_{OC} [V]	FF [%]	PCE [%]	V_{OC} × FF [V]	References
D18:Z8:L8-BO	27.2	0.92	80.8	20.2	0.743	*Nat Energy, 2024, 9: 975-986.*
Certified	27.0	0.90	81.0	19.8	0.729	
PM6:L8-BO-C$_4$:L8-BO-C$_4$-Br	27.96	0.894	81.7	20.42	0.730	*Nat Mater, 2025, 24: 433-443.*
Certified	27.76	0.889	81.2	20.1	0.722	
PM6:BTP-eC9-4ClO	27.99	0.891	80.3	20.03	0.716	*Adv. Mater. 2025, 37, 2500861*
Certified	27.42	0.894	79.3	19.45	0.709	

D18:L8-ThCl/L8-BO:L8-ThCl	27.5	0.910	80.3	20.1	0.723	*Nat Commun, 2024, 15: 6865.*
Certified	27.4	0.907	80.5	20.0	0.730	
PM6:D18:L8-BO	26.7	0.896	81.9	19.6	0.734	*Nat Mater, 2022, 21: 656–663*
Certified	26.7	0.891	80.8	19.2	0.720	
PM6:BTP-eC9:SMA	29.10	0.863	80.52	20.22	0.695	*Adv. Mater. 2024, 36, 2406690*
Certified	28.89	0.861	79.04	19.66	0.681	
PM6:PY-IT:AQI4	26.6	0.945	80.1	20.1	0.757	*J.Am.Chem.Soc.2025,147,24491−24501*
Certified	26.1	0.949	78.4	19.5	0.744	
PM6:BTP-eC9:ANF-4	29.19	0.876	79.11	20.23	0.693	*Science China Chemistry, 2025: 1-8.*
Certified	29.19	0.871	78.64	20.00	0.685	
D18 (PY-IT diluted)/L8-BO:C5-16 (D18 diluted)	27.7	0.914	82.8	21.0	0.757	*Adv. Mater. 2025, 2419923*
Certified	27.7	0.906	80.66	20.25	0.731	
D18:L8-BO:PQIC	27.3	0.926	82.3	20.8	0.762	This Work
Certified	27.46	0.917	81.79	20.6	0.750	

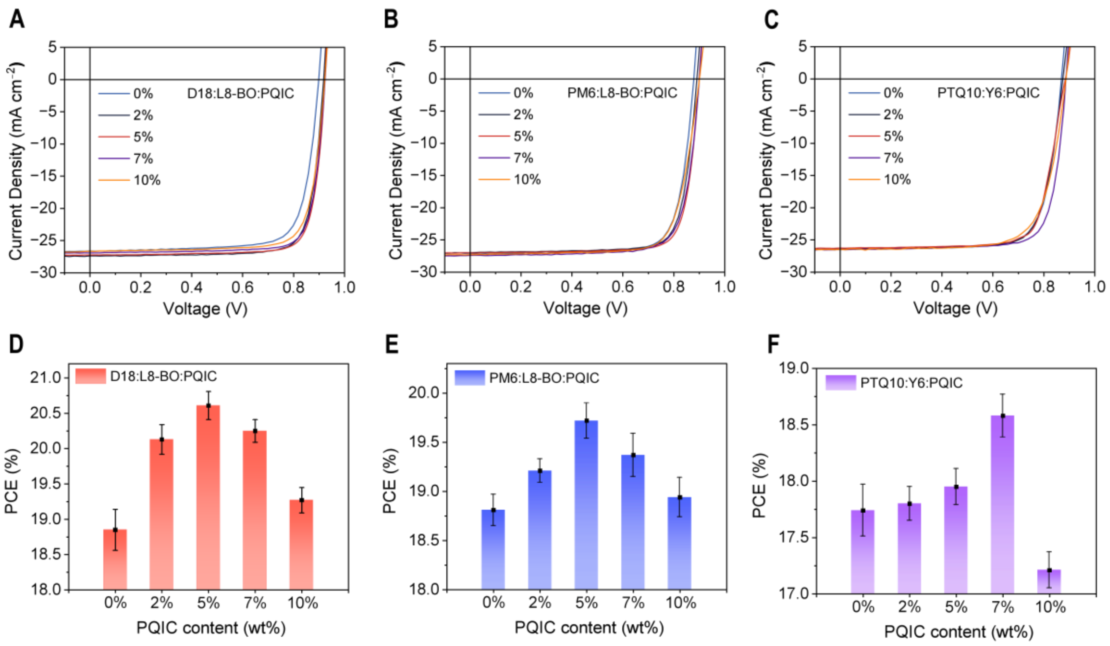

Fig. S18. *J-V* characteristics of ternary OSCs based on (**A**) D18:L8-BO:PQIC, (**B**) PM6:L8-BO:PQIC, and (**C**) PTQ10:Y6:PQIC with different PQIC contents. Corresponding bar charts of power conversion efficiencies (PCEs) for the (**D**) D18:L8-BO:PQIC, (**E**) PM6:L8-BO:PQIC, and (**F**) PTQ10:Y6:PQIC ternary devices at various PQIC loadings.

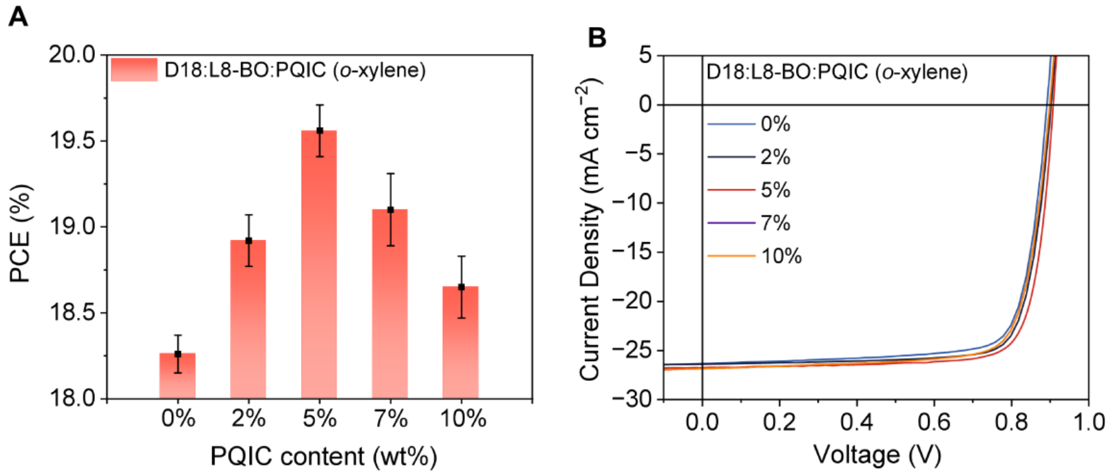

Fig. S19. (**A**) *J-V* characteristics and (**B**) corresponding power conversion efficiency (PCE) bar charts of D18:L8-BO:PQIC OSCs with varied PQIC contents, processed from *o*-xylene solvent.

Table S2. Device performances of D18:L8-BO:PQIC devices with different PQIC loading.

D18:L8-BO:PQIC	V_{OC} [V]	J_{SC} [mA cm^{-2}]	*FF* [%]	PCE [%]
0%	0.908	26.8	78.5	19.14 [18.85 ± 0.29][c]
2%	0.920	27.43	80.83	20.41 [20.13 ± 0.21][d]
5%	0.926	27.3	82.3	20.81 [20.61 ± 0.20][d]
7%	0.926	26.98	81.07	20.25 [20.01 ± 0.16][d]
10%	0.926	26.72	79.05	19.56 [19.27 ± 0.18][d]

[c] Averaged PCE values are based on 8 independent devices; [d] Averaged PCE values are based on 5 independent devices;

Table S3. Device performances of PM6:L8-BO:PQIC devices with different PQIC loading.

PM6:L8-BO:PQIC	V_{OC} [V]	J_{SC} [mA cm^{-2}]	*FF* [%]	PCE [c] [%]

0%	0.880	27.15	78.72	18.81<br>[18.63 ± 0.16][c]
2%	0.893	27.07	78.86	19.21<br>[19.04 ± 0.12][c]
5%	0.903	27.06	80.96	19.72<br>[19.52 ± 0.18][c]
7%	0.903	27.48	78.29	19.37<br>[19.11 ± 0.22][c]
10%	0.903	27.35	76.93	18.94<br>[18.73 ± 0.20][c]

[c] Averaged PCE values are based on 5 independent devices

Table S4. Device performances of PTQ10:Y6:PQIC devices with different PQIC loading.

PTQ10:Y6:PQIC	V_{OC}<br>[V]	J_{SC}<br>[mA cm^{-2}]	*FF*<br>[%]	PCE [c]<br>[%]
0%	0.871	26.57	75.87	17.74<br>[17.44 ± 0.27][c]
2%	0.876	26.52	76.27	17.80<br>[17.61 ± 0.15][c]
5%	0.887	26.32	77.48	17.95<br>[17.74 ± 0.16][c]
7%	0.889	26.53	79.61	18.58<br>[18.37 ± 0.19][c]
10%	0.889	26.54	73.67	17.21<br>[17. 02 ± 0.16][c]

[c] Averaged PCE values are based on 5 independent devices

Table S5. Device performances of D18:L8-BO:PQIC devices with different PQIC loading processed from *o*-xylene solvent.

D18:L8-BO:PQIC	V_{OC}<br>[V]	J_{SC}<br>[mA cm^{-2}]	*FF*<br>[%]	PCE [c]<br>[%]
0%	0.894	26.41	77.23	18.26<br>[18.15 ± 0.11][c]
2%	0.905	26.48	78.93	18.92<br>[18.77 ± 0.15][c]
5%	0.910	26.82	80.11	19.56<br>[19.41 ± 0.15][c]
7%	0.902	26.95	78.47	19.10<br>[18.89 ± 0.21][c]
10%	0.902	26.94	76.77	18.65<br>[18. 47 ± 0.18][c]

[c] Averaged PCE values are based on 5 independent devices

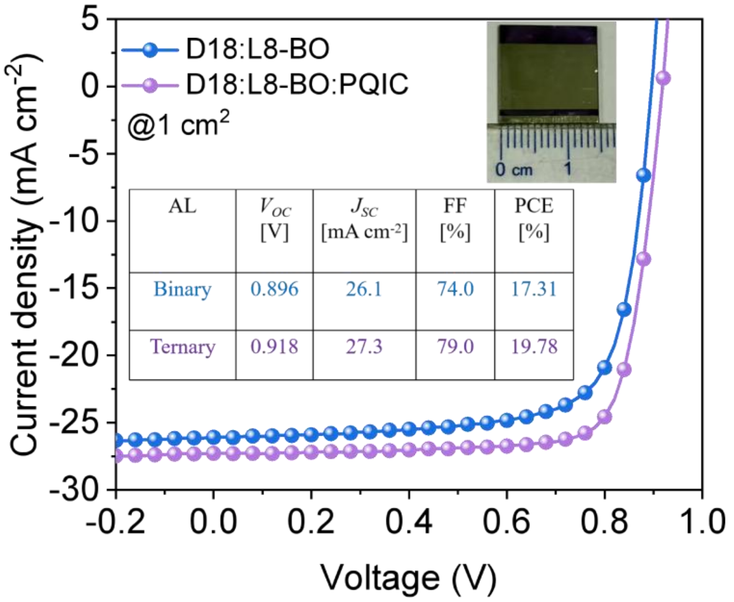

AL	V_{OC} [V]	J_{SC} [mA cm^{-2}]	FF [%]	PCE [%]
Binary	0.896	26.1	74.0	17.31
Ternary	0.918	27.3	79.0	19.78

Fig. S20. (**A**) *J-V* characteristics of binary and ternary OSCs with 1cm^2 active layer area.

J_{ph} versus V_{eff} Measurements

The photocurrent density versus effective voltage (J_{ph}–V_{eff}) curves of photovoltaic devices under different conditions were measured. Here, J_{ph} is defined as $J_{ph} = J_L - J_D$, where J_L and J_D represent the photocurrent densities under AM 1.5G solar light irradiation and in the dark, respectively. The effective voltage, V_{eff}, is calculated as $V_{eff} = V_0 - V_{appl}$, where V_{appl} is the applied bias voltage and V_0 is the voltage at which J_L equals J_D.

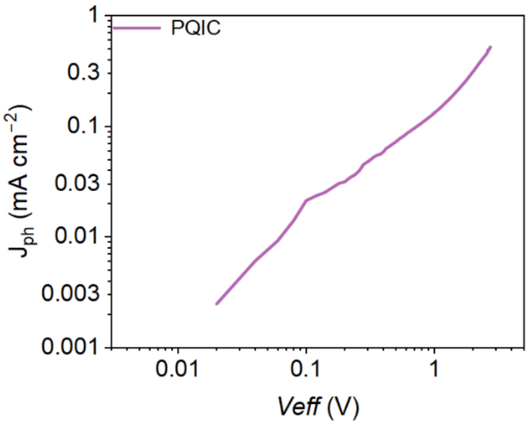

Fig. S21. Photocurrent density (J_{ph}) versus effective voltage (V_{eff}) curves for the PQIC-based OSC.

SCLC Measurements

The charge carrier mobilities of pristine and blend films are estimated from space-charge-limited current (SCLC) method. The hole-only and electron-only devices were fabricated with the architectures of ITO/PEDOT:PSS/Active layer/MoO_3/Ag and ITO/ZnO/Active layer/PFNDIT-F_3N/Ag. Hole-only and electron-only devices were recorded with a Keithley 236 source meter under dark. The hole and electron mobilities were determined by fitting the dark current to the model of single-carrier SCLC, which is described by the equation,

$$J = \frac{9}{8}\varepsilon_0\varepsilon_\gamma\mu\frac{V^2}{d^3}$$

where J is the current density, μ is the zero-field mobility, ε_0 is the permittivity of free space, ε_r is the relative permittivity of the material, d is the thickness of the active layers, and V is the effective voltage. The effective voltage was obtained by subtracting the built-in voltage (V_{bi}) and the voltage drop (V_s) from the series resistance of the whole device except for the active layers from the applied voltage (V_{appl}), $V = V_{appl} - V_{bi} - V_s$. ($V_{bi} = 0$ and $V_s = 10\times I$, where the value 10 is the resistance of MoO_3 and I is the current of the devices in this work). The hole and electron mobilities can be calculated from the slope of the $J^{1/2}$-V curves.

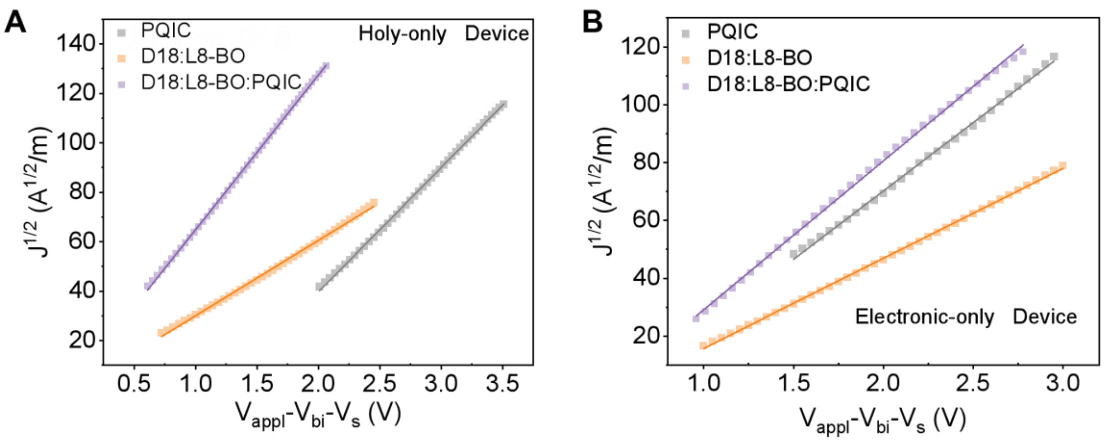

Fig. S22. SCLC results for (**A**) hole-only and (**B**) electron-only devices.

Table S6. The carrier mobilities of PQIC, D18:L8-BO binary, and D18:L8-BO:PQIC ternary films.

Devices	μ_e (cm^2 V^{-1} s^{-1})	μ_h (cm^2 V^{-1} s^{-1})	μ_h/μ_e
PQIC	2.05×10^{-3}	2.21×10^{-3}	1.08
D18:L8-BO	8.90×10^{-4}	8.43×10^{-4}	0.95
D18:L8-BO:PQIC	3.12×10^{-3}	3.48×10^{-3}	1.12

TPV and TPC Measurements

For Transient Photocurrent (TPC) measurements, a weakened 580 nm laser pulse with a pulse width of 120 fs was used as the light source. The measuring equipment was linked to a Tektronix TDS 3052C digitizing oscilloscope with a resistance of 50 Ω. For Transient Photovoltage (TPV) measurements, in order to form the open-circuit conditions, the device was connected to the oscilloscope using a high resistance of 1 MΩ. To ensure a tiny variation of the laser-pulse-induced photovoltage in this test, we used the 0.3 Sun background illumination.

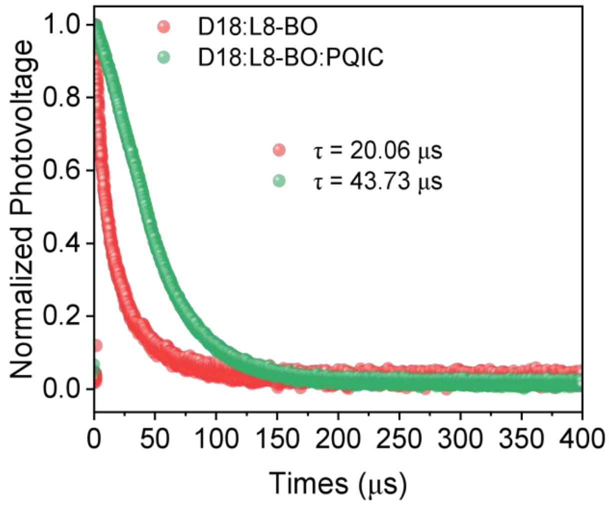

Fig. S23. TPV curves for D18:L8-BO binary and D18:L8-BO:PQIC ternary devices.

Trap Density of States (DOS) Measurements

The density of states (DOS) of the devices was determined through capacitance-frequency (C-ω) measurements. In this method, a small AC bias alternately traps and

releases carriers from states near the Fermi level, thermally exciting the trapped charges into movable transport states.

Table S7. Detailed parameters from DOS Measurements.

Devices	E_{G0} [eV]	σ [meV]	N_G [cm^{-3}]
D18:L8-BO	0.524	27.05	5.93×10^{15}
Ternary	0.501	21.3	2.91×10^{15}
PQIC	0.504	24.1	1.47×10^{15}

Energy Loss Measurements

The energy loss data were obtained by measuring the optimal OSC devices after encapsulation. The electroluminescence spectra were acquired by a high-sensitivity spectrometer (QE Pro, Ocean Optics), while the external quantum efficiency of EL was determined by measuring the emitted photons in all directions through an integrated sphere by using a calibrated spectrometer (QE Pro, Ocean Optics), with the device injected by an external current/voltage source with constant current density.

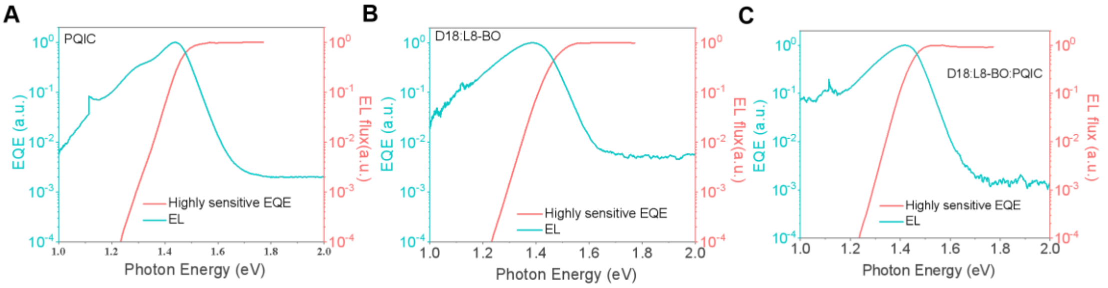

Fig. S24.The highly sensitive EQE and EL curves of (**A**) D18:L8-BO, (**B**) D18:L8-BO:PQIC and (**C**) PQIC films.

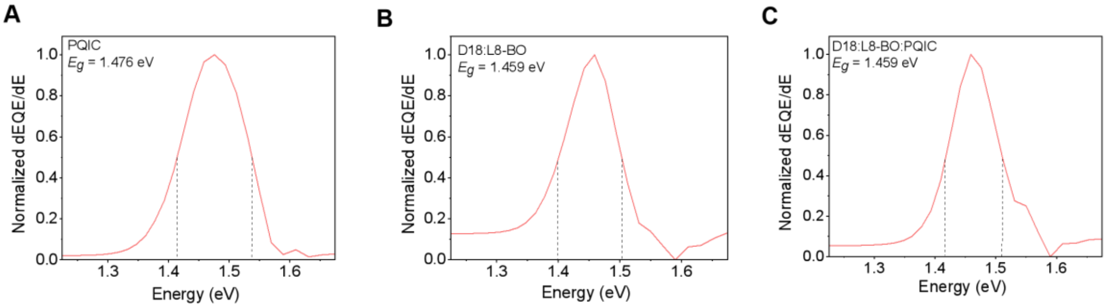

Fig. S25. Optical bandgap determination of (**A**) D18:L8-BO, (**B**) D18:L8-BO:PQIC

and (**C**) PQIC films. The bandgap was determined by differentiating the EQE spectra (dEQE/dE). The region where the gap distribution probability is greater than half of the maximum is used for the bandgap calculation.

Table S8. Detailed energy losses of the related OSCs.

Devices	E_g [eV]	E_{loss} [eV]	ΔE_1 [eV]	ΔE_2 [eV]	ΔE_3 [eV]	$qV_{OC}^{Cal.}$ [eV]
PQIC	1.476	0.620	0.259	0.207	0.154	0.856
D18:L8-BO	1.459	0.551	0.258	0.077	0.216	0.908
D18:L8-BO:PQIC	1.459	0.533	0.258	0.087	0.188	0.926

2D Grazing Incidence Wide Angle X-ray Scattering (GIWAXS)

1D GIWAXS patterns was corrected to represent real q_z and q_{xy} axes with the consideration of missing wedge. The critical incident angle was determined by the maximised scattering intensity from sample scattering with negligible contribution from underneath layer scattering. The shallow incident angle scattering was collected at 0.2°, which renders the incident X-ray as an evanescent wave along the top surface of thin films. The samples for GIWAXS test were prepared by casting solution onto silicon wafer substrates (ca. 15 mm×15 mm), and the active layers were prepared using exactly the same concentration and same procedures as those for device processing.

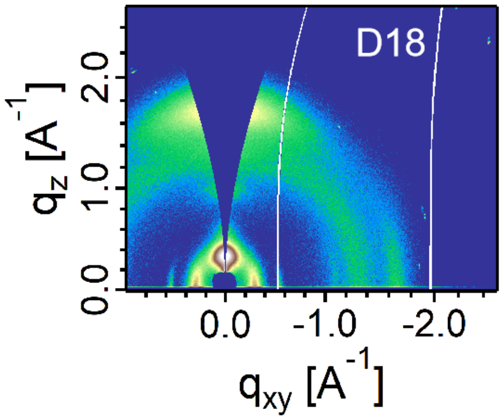

Fig. S26. 2D GIWAXS patterns of the D18 film.

Table S9. Calculated GIWAXS parameters for IP (100) peaks.

Sample	IP (100)			
	q [Å^{-1}]	*d*-spacing [Å]	FWHM [Å^{-1}]	CCL [Å]
D18	0.308	20.390	0.0775	72.082
L8-BO	0.460	13.658	0.0689	81.169
PQIC	0.242	25.950	0.0740	75.530
D18:PQIC	0.291	21.581	0.1086	51.466
L8-BO:PQIC	0.443	14.176	0.0863	64.766
D18:L8-BO	0.317	19.811	0.0706	79.168
D18:L8-BO:PQIC	0.315	19.937	0.0648	86.254

Table S10. Calculated GIWAXS parameters for IP (100) peaks.

Sample	OOP (010)			
	q [Å^{-1}]	*d*-spacing [Å]	FWHM [Å^{-1}]	CCL [Å]
D18	1.700	3.694	0.2184	25.597
L8-BO	1.764	3.560	0.3341	16.733
PQIC	1.613	3.893	0.2996	18.659
D18:PQIC	1.667	3.767	0.2921	19.139
L8-BO:PQIC	1.758	3.572	0.3036	18.414
D18:L8-BO	1.725	3.641	0.2296	24.349
D18:L8-BO:PQIC	1.727	3.636	0.2021	27.662

Photo-induced force microscope (PiFM)

The spectral linewidth is ~2 cm^{-1} with a wavenumber resolution of 0.5 cm^{-1} . The PiFM experiment here was operated at the sideband excitation with the laserfrequency modulated at f_m=f_1-f_0 , where f_0 is the first mechanical eigenmode resonances of the cantilever that is used for PiFM signal detection, while f_1 denotes the second ones recorded for the AFM topography of the sample. The probe is an Pt-coated tip with a resonant frequency of ~ 350 kHz (PPPNCHPt-MB, Nanosensors).

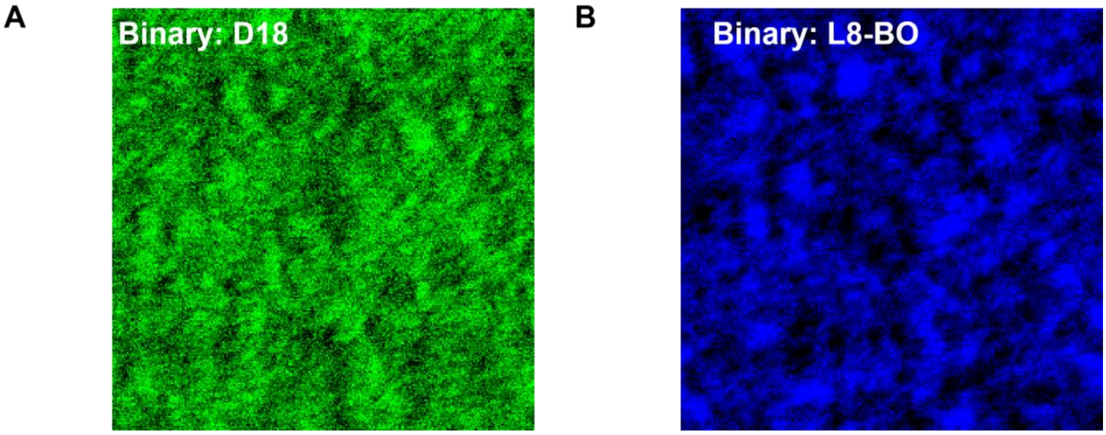

Fig. S27. PiFM images at the wavenumber of (**A**) 1772cm^{-1} (representing D18, green) and (**B**) 1422 cm^{-1} (representing L8-BO, Blue) for the D18:L8-BO blend films.

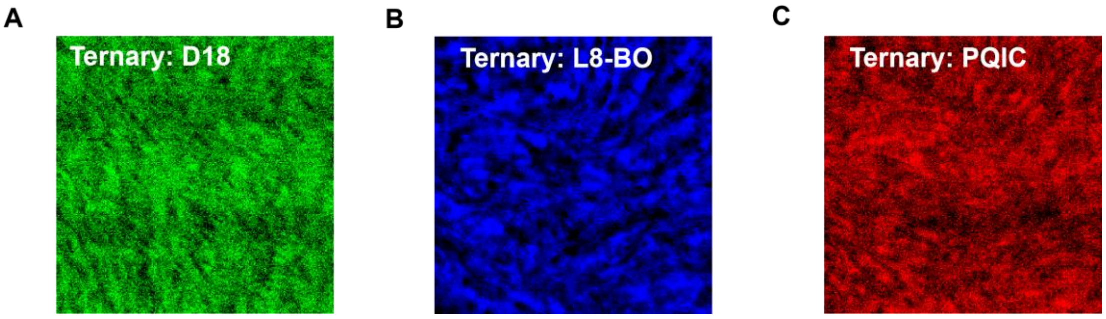

Fig. S28. PiFM images at the wavenumber of (**A**) 1772cm^{-1} (representing D18, green), (**B**) 1422 cm^{-1} (representing L8-BO, Blue) and (**C**) 1363 cm^{-1} (representing PQIC, red) for the ternary films .

Temperature-dependent Photoluminescence (PL) Measurements

Photoluminescence (PL) measurements were performed on active-layer films deposited on quartz substrates. The films were mounted on a thermally conductive copper stage and cooled to 100 K using liquid nitrogen. A 660 nm laser was employed as the excitation source, with its intensity and position kept constant throughout the experiment. PL spectra were then recorded at every 40 K interval using a high-sensitivity spectrometer (QE Pro, Ocean Optics).

Temperature-dependent Absorption Measurements

For temperature-dependent absorption spectroscopy, thin-film samples were prepared by spin-coating chloroform solutions onto quartz substrates, yielding films with a

thickness of approximately 100 nm. The films were mounted in an optical cryostat (Wentian Jingce CH190-400) using thermally conductive tape to ensure efficient thermal contact with the copper cold stage. Temperature control was achieved by cooling the stage with liquid nitrogen. At each target temperature, the samples were allowed to stabilize for 60 s prior to measurement. A halogen light source (ORITEX D100LR) was used for illumination, with both the light intensity and optical alignment maintained constant throughout the experiment. Absorption spectra were collected at 10 K intervals using a high-sensitivity spectrometer (QE Pro, Ocean Optics).

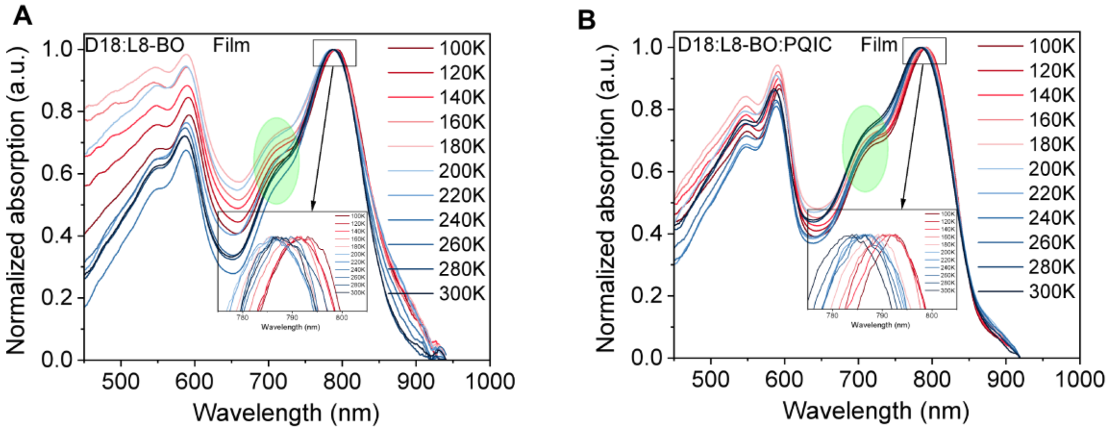

Fig. S29. The normalized temperature-dependent absorption spectra of D18:L8-BO binary, and D18:L8-BO:PQIC films.

Temperature-dependent Deep-level Transient Spectroscopy (DLTS) Measurements

Temperature-dependent deep-level transient spectroscopy (DLTS) measurements were performed using the transient photocurrent method. The samples were fabricated using standard device fabrication processes. During the measurements, the sample was cooled using liquid nitrogen. A bias of 0 V was first applied to populate the traps within the space charge region. Subsequently, upon switching to a reverse bias, the filled traps could release the captured charge carriers as the temperature was increased to a new bias condition. The carrier emission process manifests as a transient current in the device current signal. Based on the experimental data, the areal density of trap states, N_t, can be calculated using the following formula:

$$j_{te}(t) = \frac{1}{\tau_{te}} \cdot q \cdot d \cdot N_t \cdot exp(-\frac{1}{\tau_{te}})$$

Here, j_{te} is trap emission current, τ_{te} is catch-trap emission time constant, q is a single charge amount, d is the thickness of the device.

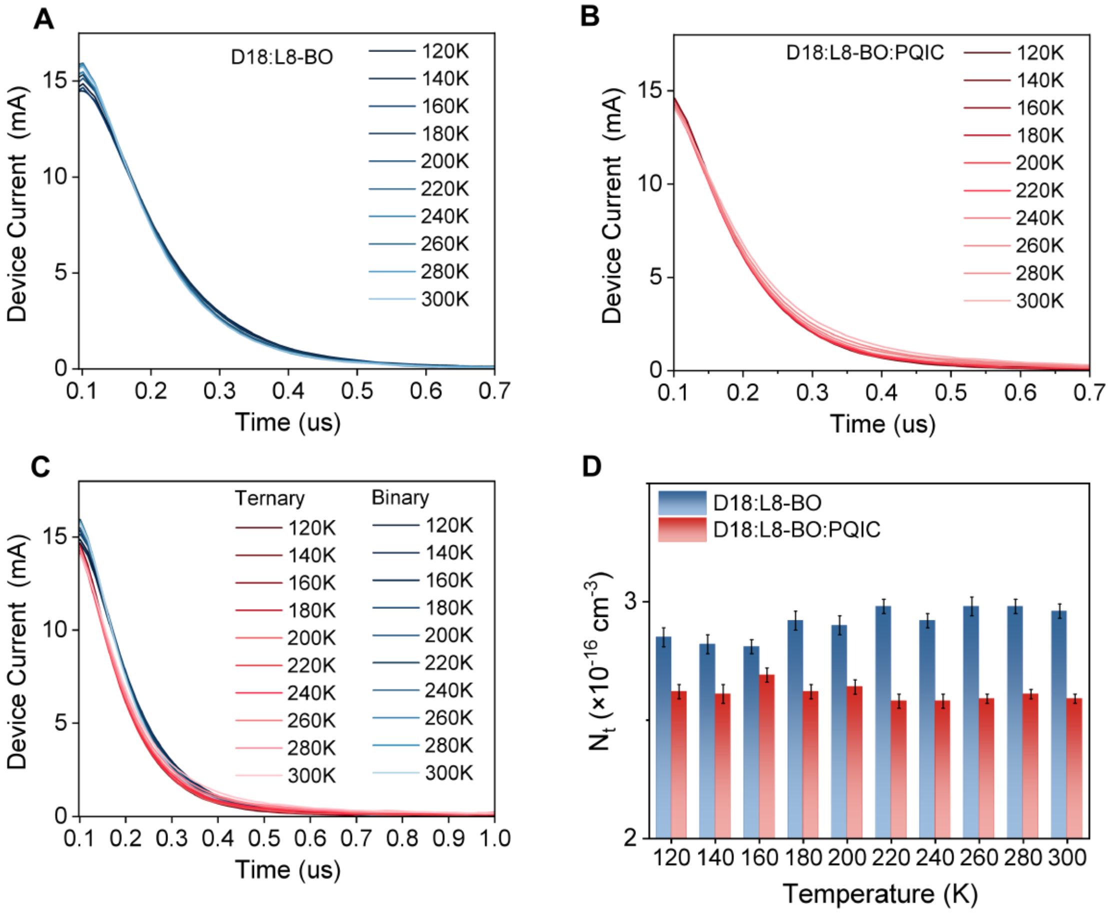

Fig. S30. (**A-C**) In situ DLTS measurements (**D**) and the derived defect-state density of binary and ternary films at various temperatures.

Ultra-fast TAS Measurements

Ultrafast laser pulses (800 nm, < 35 fs pulse duration, 7 W) was generated by 1 kHz Ti:Sapphire regenerative amplifier (Astrella, Coherent, USA). 2.4 W of the fundamental pulses (7 W) was used to pump the commercial collinear optical parametric amplifier (TOPAS Prime, Light-Conversion, Lithuania). The pump beam is chopped at 500 Hz. 15% of the fundamental pulses was routed onto a mechanical delay stage (within 7 ns) and passed through a sapphire crystal to generate supercontinuum probe light (450-750 nm). The pump light and probe light were focused on a same spot

(2 mm diameter) of the thin films spin-coating on a quartz. Data analysis is performed by Surface Xplorer software. The incident power is measured with a calibrated laser power meter.

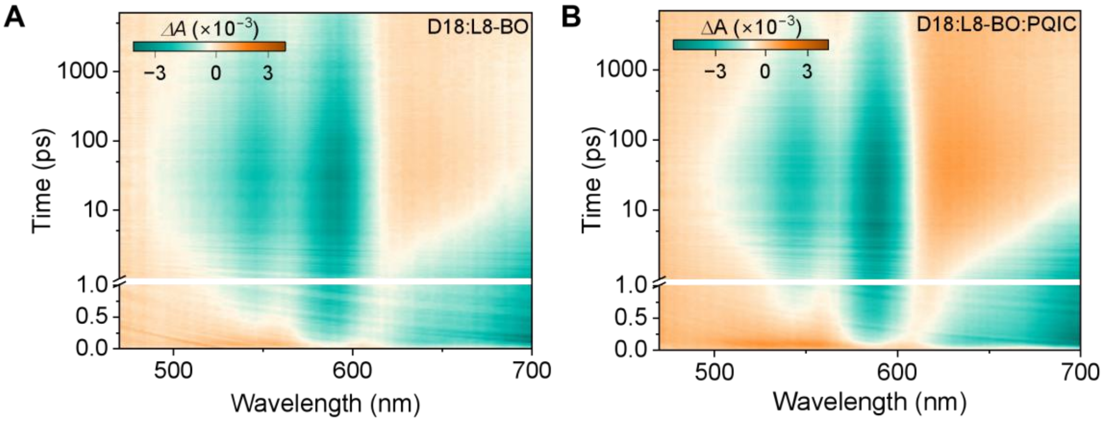

Fig. S31. 2D contour maps of fs-TAS results for (**A**) D18:L8-BO binary and (**B**) D18:L8-BO:PQIC ternary devices.

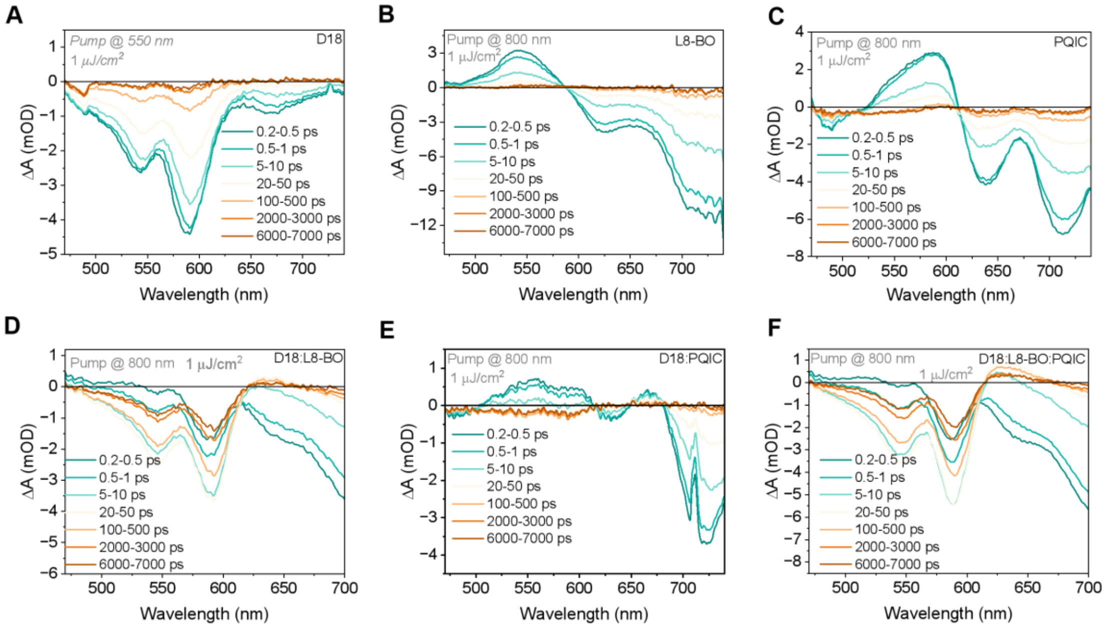

Fig. S32. The time selected spectra of neat (**A**,**B**,**C**) and blend films (**D**,**E**,**F**).

Stability Measurements

The testing devices were fabricated under the same preparation conditions as those used for the *J–V* curve measurements and were not further encapsulated. Light-induced degradation experiments were conducted under continuous one-sun equivalent

illumination. The devices were placed inside a nitrogen-filled glovebox (H_2O < 0.01 ppm, O_2 < 0.03 ppm) and mounted on a thermally conductive copper plate. An insulating glass sheet was inserted between the copper plate and the device to provide a stable thermal environment via heat conduction. During the aging process, the devices were continuously illuminated and maintained at their maximum power point. The *J–V* characteristics were periodically measured, and the photovoltaic parameters were automatically calculated based on the acquired *J–V* curves. All *J–V* measurements were performed using a computer-controlled Keithley 2450 source meter under a calibrated solar simulator with an illumination intensity of 100 mW cm^{-2}. The light intensity was verified prior to testing using a calibrated silicon photodiode to ensure accuracy.

Deviation Matrix (DMT) Method Measurements

The unannealed film was placed on a quartz substrate, and its absorption spectrum was first recorded at 30 °C. After the initial measurement, the film was heated on a temperature-controlled heating stage at 40 °C for 2 min, removed from the stage, allowed to cool to room temperature, and then measured. The same procedure was subsequently carried out at higher temperatures: the film was heated at 50 °C for 2 min, cooled, and measured. This stepwise annealing–cooling–measurement cycle was repeated with temperature increments of 10 °C.

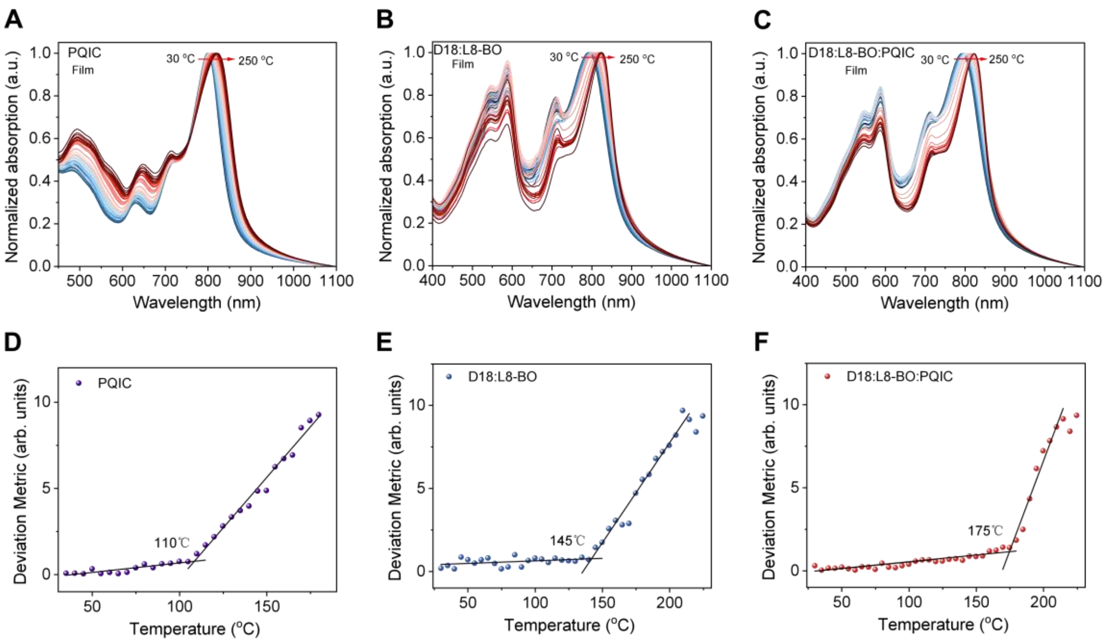

Fig. S33. Normalized absorption spectra of (**A**) PQIC, (**B**) D18:L8-BO, and (**C**) D18:L8-BO:PQIC films after thermal annealing at different temperatures for 2 min, with the temperature increased in 10 ºC intervals. Deviation Matrix Method (DMT) results of (**D**) PQIC, (**E**) D18:L8-BO, and (**F**) D18:L8-BO:PQIC films.
